# Biomimetic Liposomal Nanoplatinum for Targeted Cancer Chemophototherapy

**DOI:** 10.1002/advs.202003679

**Published:** 2021-03-01

**Authors:** Xue‐Liang Liu, Xiao Dong, Si‐Cong Yang, Xing Lai, Hai‐Jun Liu, Yuhao Gao, Hai‐Yi Feng, Mao‐Hua Zhu, Yihang Yuan, Qin Lu, Jonathan F. Lovell, Hong‐Zhuan Chen, Chao Fang

**Affiliations:** ^1^ Hongqiao International Institute of Medicine Tongren Hospital and State Key Laboratory of Oncogenes and Related Genes Department of Pharmacology and Chemical Biology Shanghai Jiao Tong University School of Medicine Shanghai 200025 China; ^2^ Department of Biomedical Engineering University at Buffalo State University of New York Buffalo NY 14260 USA; ^3^ Institute of Interdisciplinary Integrative Biomedical Research Shuguang Hospital Shanghai University of Traditional Chinese Medicine Shanghai 201203 China

**Keywords:** biomimetics, liposomes, photodynamic therapy, platinum nanoparticles, tumor penetration

## Abstract

Photodynamic therapy (PDT) of cancer is limited by tumor hypoxia. Platinum nanoparticles (nano‐Pt) as a catalase‐like nanoenzyme can enhance PDT through catalytic oxygen supply. However, the cytotoxic activity of nano‐Pt is not comprehensively considered in the existing methods to exert their multifunctional antitumor effects. Here, nano‐Pt are loaded into liposomes via reverse phase evaporation. The clinical photosensitizer verteporfin (VP) is loaded in the lipid bilayer to confer PDT activity. Murine macrophage cell membranes are hybridized into the liposomal membrane to confer biomimetic and targeting features. The resulting liposomal system, termed “nano‐Pt/VP@MLipo,” is investigated for chemophototherapy in vitro and in vivo in mouse tumor models. At the tumor site, oxygen produced by nano‐Pt catalyzation improves the VP‐mediated PDT, which in turn triggers the release of nano‐Pt via membrane permeabilization. The ultrasmall 3–5 nm nano‐Pt enables better penetration in tumors, which is also facilitated by the generated oxygen gas, for enhanced chemotherapy. Chemophototherapy with a single injection of nano‐Pt/VP@MLipo and light irradiation inhibits the growth of aggressive 4T1 tumors and their lung metastasis, and prolongs animal survival without overt toxicity.

## Graphical abstract

The biomimetic liposomal nano‐Pt (nano‐Pt/VP@MLipo) targets the tumor sites, where oxygen produced by nano‐Pt catalyzation enhances the verteporfin (VP)‐mediated photodynamic therapy (PDT). PDT in turn permeabilizes the liposome membrane for efficient nano‐Pt release. These ultrasmall particles (3–5 nm) achieve penetration in deeper tumor tissue, which is also facilitated by the generated oxygen gas, for enhanced chemotherapy.

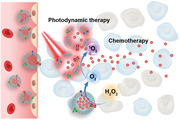

## 1 Introduction

Photodynamic therapy (PDT) is a clinically approved, site‐specific, minimally invasive therapy for solid tumors.^[^ ^1^ ^]^ The anti‐tumor effect of PDT depends on the spatiotemporal cooperation of photosensitizer, molecular oxygen (O_2_) and laser with specific wavelength and intensity. Through photochemical reactions of the light‐activated photosensitizer and O_2_, singlet oxygen (^1^O_2_) is produced that exerts therapeutic effects. However, in general, the heterogenous solid tumor microenvironment itself is already hypoxic in some locations.^[^ ^2^ ^]^ Hypoxia compromises the efficacy of oxygen‐dependent PDT, and presents as a reason for incomplete tumor killing and disease recurrence when PDT is performed alone.^[^ ^1^ ^]^ Thus, the combination of PDT with other therapeutic intervention, such as chemotherapy (called chemophototherapy), for improved outcome is intriguing and received recent attention.^[^ ^3^ ^]^ In fact, nanotechnology‐based multimodal synergistic therapy is emerging as a promising strategy to combat cancer with high efficacy.^[^ ^1^, ^4^ ^]^

To overcome the hypoxia bottleneck, researchers have developed a variety of methods to reduce tumor hypoxia and enhance the PDT efficacy. One strategy is by delivering perfluorocarbons (PFCs) carrying exogenous oxygen for enhanced PDT.^[^ ^5^, ^6^ ^]^ However, the side effects of PFC, when translated into humans, has including decrease in arterial pressure, lung damage, thrombocytopenia, and flu‐like symptoms, as well as the dosage form‐associated stability concerns should be carefully considered.^[^ ^7^ ^]^ The other is generating oxygen through decomposition of H_2_O_2_, which is commonly elevated to a higher level in cancer cells compared to normal cells.^[^ ^8^ ^]^ For this purpose, nanoparticle‐based targeted delivery of catalase (CAT) has been introduced, and thereby relieving tumor hypoxia for improved PDT.^[^ ^9^ ^]^ However, as a 240 kDa biomacromolecule, poor stability and difficulty in loading and delivering across biological barriers may hinder the wide application.^[^ ^10^ ^]^

Recently, nanozymes (nanomaterials with enzyme‐like characteristics) with the features of low cost, good stability, and mass‐production have been developed to address limitations of natural enzymes.^[^ ^11^ ^]^ Moreover, nanozymes may also possess additional multifunctional properties, such as imaging capability. Several CAT‐like nanozymes have been developed, including those based on MnO_2_^[^ ^12^ ^]^ and platinum,^[^ ^13^ ^]^ which have been demonstrated as oxygen‐replenishing nanomaterials.

Platinum nanoparticles (nano‐Pt) also have potent cytotoxicity, and can serve as a chemotherapeutic agent, through the leaching of Pt ions.^[^ ^14^, ^15^ ^]^ However, to the best of our knowledge, previous research reports have focused on either the CAT‐mimicry, or chemotherapy potency of nano‐Pt only, with few efforts exploiting their dual activities for cancer therapy. Specifically, for typical loading, nano‐Pt was tethered on the nanocarriers using the in situ growth method.^[^ ^13^ ^]^ Due to the limitation of the carrier dimension (90–130 nm), ^[^ ^13^ ^]^ the chemotherapy potency of nano‐Pt might be constrained, with less impact on the tumor cells in the deeper tumor parenchyma further away from blood vessels from which they extravasate.

**Scheme 1:**
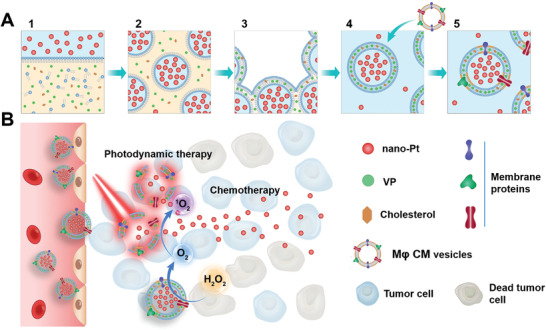
Schematic illustration of the fabrication A) of nano‐Pt/VP@MLipo and B) chemophototherapy performance in tumors. 1) Lipid and VP was dispersed in organic solvent (light yellow), and nano‐Pt was introduced in the water (light blue). 2) The mixture was sonicated to from an emulsion. 3) Organic solvent was evaporated until the gel‐like state was disappeared. 4) PBS addition led to the formation of liposomes containing nano‐Pt in the water cavity and VP in the lipid bilayer. M*φ* cell membrane (CM) vesicles were then added for follow‐up hybridization. 5) The mixture was then subjected to freeze–thaw cycles and extrusion to generate the final biomimetic nano‐Pt/VP@MLipo prior to removal of unentrapped components.

In this work, we developed a new nano‐Pt delivery strategy, which exploited both the CAT‐like activity and the chemotherapy potency to achieve the synergistic chemophototherapy (**Scheme** **1**). It is a challenge to not only keep nano‐Pt free, thus not be restricted by the conventional in situ growth method, for tumor penetration and meanwhile obtain high loading with their ultrasmall dimension (3–5 nm) and hydrophilicity. We successfully encapsulated nano‐Pt in the inner aqueous cavity of liposomes by the reverse phase evaporation technique.^[^ ^16^ ^]^ The lipid bilayer was used to load the hydrophobic, clinical photosensitizer verteporfin (VP). The resulting nano‐Pt/VP@Lipo was then hybridized with RAW264.7 macrophage (M*φ*) cell membrane (CM) to generate nano‐Pt/VP@MLipo. Camouflage with M*φ* membrane proteins is expected to endow the liposome with biomimetic properties, such as long circulation and inflammatory endothelium (e.g., tumor vessel) targeting.^[^ ^17^, ^18^ ^]^ After intravenous (i.v.) injection, nano‐Pt/VP@MLipo would home to the tumor site, where nano‐Pt catalyzes the decomposition of high level of H_2_O_2_, provides oxygen, and thus enhances the VP‐based PDT effect. ^1^O_2_ produced in PDT will also disrupt the liposomal membrane ^[^ ^19^, ^20^, ^21^ ^]^ and liberate the ultra‐small nano‐Pt for improved tumor penetration and chemotherapy.^[^ ^22^ ^]^ The physicochemical characteristics are investigated and the chemophototherapy efficacy of nano‐Pt/VP@MLipo in vitro and in vivo is demonstrated.

## 2 Results and Discussion

### 2.1 Preparation and Characterization of Nano‐Pt/VP@MLipo

**Figure 1:**
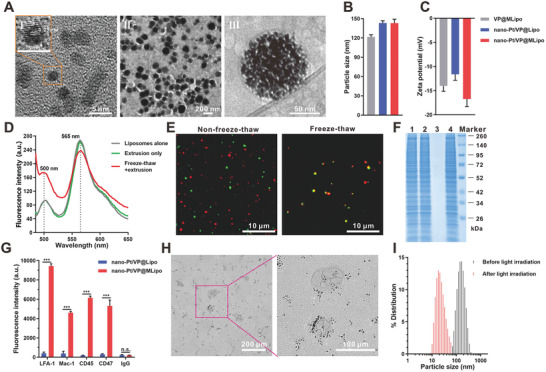
Characterizations of nano‐Pt/VP@MLipo. A) TEM images of free I) nano‐Pt and II) nano‐Pt/VP@MLipo. III) An enlarged Cryo‐TEM image of nano‐Pt/VP@MLipo. B) Particle size and C) zeta potential measured through dynamic light scattering (DLS). D) Liposomes were labeled with DiO‐DiI FRET pair. The recovered fluorescence of DiO at ≈500 nm and meanwhile weaken FRET signal at 565 nm indicated the fusion of liposomes and M*φ* CM vesicles. E) CLSM images of the mixture of M*φ* CM vesicles (DiO, green) and liposomes (DiR, red) subjected to freeze–thaw process or not. F) SDS‐PAGE of the samples with equivalent protein contents (25 µg). 1) M*φ* CM; 2) MLipo (empty); 3) nano‐Pt/VP@Lipo; 4) nano‐Pt/VP@MLipo. G) Flow cytometry validation of the functional proteins (LFA‐1, Mac‐1, CD45, and CD47) and their orientation on the liposome surface. H) TEM observation of the light (690 nm) irradiation‐triggered nano‐Pt liberation from the aqueous cavity of nano‐Pt/VP@MLipo. I) Light irradiation‐induced size change identified using DLS. Data are presented as mean ± s.d. (*n* = 3). ***p < 0.001.

Nano‐Pt was prepared through one‐step reduction of H_2_PtCl_6_ by NaBH_4_ in the presence of folic acid, a capping agent that can stop the growth of platinum nanoparticles and stabilize them from aggregation.^[^ ^23^ ^]^ Nano‐Pt was ultrasmall with a diameter of 3–5 nm, and the 0.2 nm lattice spacing was clearly visible under transmission electron microscopy (TEM) (**Figure** **1**A‐I), which was consistent with that reported previously.^[^ ^24^ ^]^

For nano‐Pt encapsulation, the reverse phase evaporation technique, but not thin film hydration method, was adopted, which can confer high internal aqueous loading (such as nano‐Pt in this study) especially for water soluble components (Figure S1A, Supporting Information).^[^ ^16^ ^]^ Hydrophobic VP was added into the phospholipid lipid‐contained organic phase for coencapsulation. The resulting nano‐Pt and VP coloaded crude liposomes were then hybridized with M*φ* cell membrane (CM) vesicles through 5 freeze–thaw cycles at 0 and 45 °C, ^[^ ^17^, ^25^ ^]^ and then subjected to extrusion using Avanti miniextruder to generate the M*φ*‐biomimetic nano‐Pt/VP@MLipo. Compared to surface PEGylation, biomimetic membrane camouflage can potentially avoid the problems of accelerated blood clearance (ABC) caused by the generated anti‐PEG IgM and activated complements during multiple dosing.^[^ ^26^ ^]^ Moreover, functional proteins from the M*φ* CM, such as LFA‐1 and Mac‐1, have been proposed to confer inflammatory endothelium (such as tumor vessels) targeting property. ^[^ ^17^, ^18^ ^]^

The dark brown color of the resulting nano‐Pt/VP@MLipo was derived from the loaded nano‐Pt, and the yellow green color from VP was also faintly visible (Figure S2, Supporting Information). Nano‐Pt/VP@MLipo can be collected and purified through centrifugation (12 000 rpm, 10 min), while the unencapsulated, free nano‐Pt remained in the supernatant (Figure S1B, Supporting Information). Platinum and phosphorus were identified in energy‐dispersive X‐ray spectroscopy (EDS), indicting the specific element compositions of the hybrid liposomes encapsulating nano‐Pt (Figure S3, Supporting Information). Nano‐Pt/VP@MLipo was spherical and well monodispersed with ≈120 nm diameter in TEM, and were gray to black due to the strong electron absorption by the loaded platinum nanoparticles (Figure 1A‐II). The encapsulated nano‐Pt and the liposome membrane structure were distinguishable in the Cryo‐TEM image (Figure 1A‐III).

Hydrodynamic size of nano‐Pt/VP@MLipo was ≈140 nm measured by dynamic light scattering (Figure 1B). Zeta potential of nano‐Pt/VP@MLipo was −16.7 mV, higher than that (−11.6 mV) of conventional nano‐Pt/VP@Lipo without CM hybridization, exhibiting the neutralization result between the components of lipid and CM vesicles (−23.4 mV) (Figure 1C; Table S1, Supporting Information). The encapsulation efficiencies (EE%) of platinum detected by inductively coupled plasma‐optical emission spectrometry (ICP‐OES) and VP measured by fluorescence assay were high with 48.2% and 89.5%, respectively. The drug loading (DL%) of platinum in nano‐Pt/VP@MLipo was ≈15%, higher than the commercial liposomal doxorubicin (Doxil, doxorubicin loading ≈10%) engineered through the classic transmembrane pH gradient method.^[^ ^27^ ^]^ VP loading was determined to be ≈0.87%. The freeze–thaw process did not cause leakage of both cargoes as proved by the comparable EE% (Table S1, Supporting Information). It was reported that freeze–thaw cycles were even beneficial, in some circumstances, for the encapsulation of small particles in the aqueous cavity of liposomes.^[^ ^21^ ^]^

The fusion of liposome and M*φ* CM was verified using the Forster resonance energy transfer (FRET) test. The liposomes were previously labeled with both 3,3′‐dioctadecyloxacarbocyanine perchlorate (DiO) (FRET donor) and 1,1′‐dioctadecyl‐3,3,3′,3′‐tetramethylindocarbocyanine perchlorate (DiI) (FRET acceptor), and then mixed with M*φ* CM. After freeze–thaw cycles, the recovered fluorescence of DiO at 500 nm and meanwhile weaken FRET signal at 565 nm indicated the interspersing of the two membrane materials (Figure 1D). However, without freeze–thaw process, FRET intensity remained unchanged, indicating no membrane fusion occurred.

The membrane fusion was also confirmed through the observation under confocal laser scanning microscopy (CLSM). For this assay, M*φ* CM vesicles and liposomal membrane were labeled with DiO (green) and 1,1′‐dioctadecyl‐3,3,3′,3′‐tetramethylindotricarbocyanine iodide (DiR) (red), respectively. Good colocalization (yellow) of fluorescent signals was observed for the prepared MLipo (empty) (Figure 1E; Figure S4, Supporting Information). In contrast, the mixture of liposomes and M*φ* CM without freeze–thaw exhibited separate green and red dotty signals. In this assay, DiR, not DiI, was used for liposome labeling to avoid the FRET influence (Figure 1D), which may compromise the signal from M*φ* CM.

Sodium dodecyl sulfate polyacrylamide gel electrophoresis (SDS‐PAGE) was used to identify the protein profile of the liposomes. It showed hybrid membrane from MLipo (empty) or nano‐Pt/VP@MLipo well retained M*φ* proteins (Figure 1F), indicating the successful fusion of liposomes and M*φ* CM.

Using flow cytometry assay, the representative M*φ* CM proteins (LFA‐1, Mac‐1, CD45, and CD47) were identified to be present with the correct orientation on the surface of nano‐Pt/VP@MLipo (Figure 1G; Figures S5 and S6, Supporting Information). LFA‐1 and Mac‐1 are relative to the leukocyte adhesion to inflamed endothelium, which may contribute liposomal tumor targeting. CD45 is the common antigen of leukocytes. CD47 is the “marker‐of‐self” protein, which would confer long‐circulation feature for the liposomes in vivo.

It has been shown that photosensitization from the membrane‐contained photosensitizers can induce increased membrane permeability to release encapsulated cargo.^[^ ^19^, ^21^ ^]^ As expected, 690 nm light irradiation triggered liberation of nano‐Pt from nano‐Pt/VP@MLipo, which was confirmed by TEM (Figure 1H). Moreover, size change (shrinkage) after light irradiation, as identified by dynamic light scattering (DLS) assay indicated the disruption of the liposome integrity and the leakage of nano‐Pt (Figure 1I). Without VP in the formulation, light irradiation did not affect the size of nano‐Pt@MLipo as identified using DLS (Figure S7, Supporting Information). These results indicated that nano‐Pt release depended on the VP‐mediated PDT. Liberated ultrasmall nano‐Pt (3–5 nm) can then further penetrate into deeper tumor tissue for improved chemotherapy. Nano‐Pt/VP@MLipo maintained relatively good colloid stability in fetal bovine serum (FBS) and mouse serum at 37 °C for 24 h, and in PBS at 4 °C for 7 days (Figures S8–S10, Supporting Information). In addition, lipid dissociation, another parameter reflecting the stability of liposomes in serum, needs to be examined further.^[^ ^28^ ^]^

### 2.2 Nano‐Pt Catalyzed H_2_O_2_ Decomposition for O_2_ Production and Improved Reactive Oxygen Species (^1^O_2_) upon Light Irradiation

**Figure 2:**
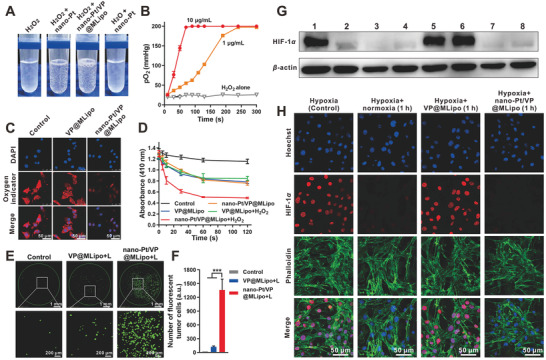
Nano‐Pt/VP@MLipo catalyzed H_2_O_2_ decomposition for O_2_ production, alleviated hypoxia and HIF‐1*α* expression in 4T1 cells and elevated the reactive oxygen species (ROS, ^1^O_2_) levels upon light (690 nm) irradiation. A) H_2_O_2_ (100 × 10^−6^ m) was decomposed by nano‐Pt catalyzation. The generated O_2_ bubbles were seen adsorbed on the inside of the tube wall. Pure water containing free nano‐Pt was set as a negative control. B) Partial oxygen pressure (pO_2_) with time after adding nano‐Pt/VP@MLipo (1 or 10 µg mL^−1^ Pt) into H_2_O_2_ solution. C) Nano‐Pt increased the intracellular oxygenation as examined using the oxygen indicator [Ru(dpp)_3_]Cl_2_. The red fluorescence of the dye was quenched in the presence of oxygen. D) ^1^O_2_ production determined using DPBF assay. DPBF was oxidized by ^1^O_2_, and led to a decreased absorbance at 410 nm. E) Intracellular ROS production upon light (690 nm) irradiation detected using the probe DCFH‐DA. The fluorescent 4T1 cells in the 96‐well plate were monitored and photographed using IncuCyte live cell analysis system. F) The number of fluorescent cells in panel (E) were quantified. G) Western blot of HIF‐1*α* expression in 4T1 cells after various treatments. 1) Hypoxia (Control); 2) Normoxia; 3) Hypoxia + normoxia (1 h); 4) Hypoxia + normoxia (4 h); 5) Hypoxia + VP@MLipo (1 h); 6) Hypoxia + VP@MLipo (4 h); 7) Hypoxia + nano‐Pt/VP@MLipo (1 h); 8) Hypoxia + nano‐Pt/VP@MLipo (4 h). H) CLSM observation of HIF‐1*α* expression and its nuclear translocation in 4T1 cells. The F‐actin was labeled with FITC‐labeled phalloidin to outline the cells. Data are presented as mean ± s.d. (*n* = 3). ***p < 0.001.

H_2_O_2_ is produced at increased levels in tumors, which can be used as a source for O_2_ production using CAT‐mimetics. Oxygen bubbles produced by nano‐Pt (either free or encapsulated in the liposomes) were visible by eye upon mixing H_2_O_2_ (**Figure** **2**A). This O_2_ production was also directly measured using an Oxylite fiber‐optic oxygen sensor (Oxford Optronix). The oxygen partial pressure (pO_2_) increased rapidly in a Pt concentration‐dependent manner, and reached the maximum value (200 mmHg) that can be detected by the instrument, exhibiting potent O_2_ production capacity (Figure 2B). It is noted that for normal tissues, pO_2_ ranged from 9 to 100 mmHg.^[^ ^29^ ^]^ However, H_2_O_2_ alone was stable with no obvious O_2_ release.

O_2_ production in tumor cells via nano‐Pt catalyzation was also examined. The test was performed under hypoxic conditions in a Captair Pyramid (Erlab) with pO_2_ below 5 mmHg. The fluorescence signal (red) of the oxygen probe [Ru(dpp)_3_]Cl_2_ was largely quenched in cancer cells treated with nano‐Pt/VP@MLipo, indicating the pronounceable intracellular O_2_ generation (Figure 2C). This effect was also Pt concentration dependent (Figure S11, Supporting Information). In contrast, the fluorescent signal remained nearly unchanged if no nano‐Pt was contained in the liposomes.

This elevated O_2_ level through H_2_O_2_ decomposition would be helpful for subsequent generation of singlet oxygen (^1^O_2_) with light irradiation of VP. 1,3‐Diphenylisobenzofuran (DPBF), which can be oxidized by ROS and led to a declined absorption at 410 nm, was adopted as the probe for ^1^O_2_ detection. The test was also performed under hypoxia. Compared to VP@MLipo, the rapidly and more decreased DPBF absorbance was observed for nano‐Pt/VP@MLipo in the presence of same light irradiation and H_2_O_2_ exposure, indicating the mass production of ^1^O_2_ via the nano‐Pt‐catalyzed O_2_ generation (Figure 2D). H_2_O_2_ alone without nano‐Pt involvement cannot lead to more ^1^O_2_ production (VP@MLipo vs VP@MLipo+ H_2_O_2_), indicating the essential role of nano‐Pt.

The increased ROS levels in tumor cells (4T1) conferred by nano‐Pt were also examined. DCFH‐DA, as an intracellular ROS fluorescent probe, was used for this test. Much more fluorescent tumor cells were observed after the cells were treated with nano‐Pt‐contained liposomes, indicating that nano‐Pt promoted the production of more ROS under light irradiation (Figure 2E,F; Figure S12, Supporting Information).

Hypoxia‐inducible factor‐1*α* (HIF‐1*α*) is a core regulator for adapting to cellular oxygen levels, and transcriptionally activates genes for oxygen homeostasis and metabolism modulation. ^[^ ^30^ ^]^ In view of this, the expression of HIF‐1*α* was negatively correlated with intracellular oxygen content. We investigated the effect of nano‐Pt loaded in liposomes on the expression of HIF‐1*α* in hypoxic 4T1 cells using Western blot. HIF‐1*α* expression was significantly reduced by liposomes containing nano‐Pt after 1 or 4 h incubation, which was comparable to that under normoxic culture (Figure 2G). Moreover, this effect is equivalent to that of restoring hypoxic tumor cells to normoxic culture for 1 or 4 h.

HIF‐1*α* expression in cells was also directly examined under CLSM. Under hypoxia, highly expressed HIF‐1*α* was dominantly translocated into the cell nuclei for adaptive metabolism response (Figure 2H; Figure S13, Supporting Information). However, the expression was almost completely abolished when the hypoxic cells were treated with nano‐Pt‐contained liposomes for only 1 h, or returned to normoxic culture. The CLSM observation for HI1‐1*α* expression was well consistent with the Western blot results.

### 2.3 Cytotoxicity of Nano‐Pt/VP@MLipo with Light Irradiation and Nano‐Pt

**Figure 3:**
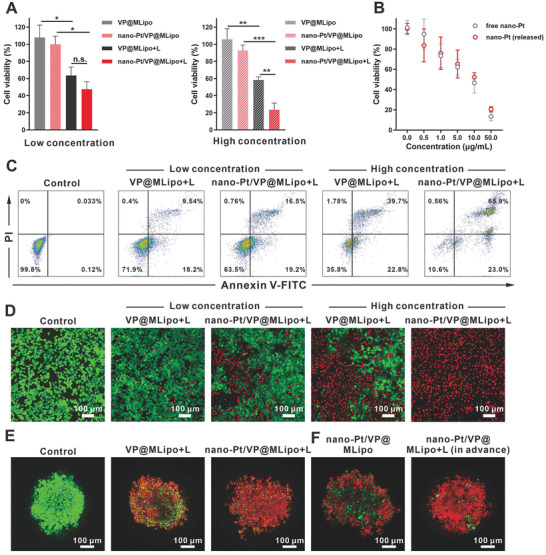
Cytotoxicity of nano‐Pt/VP@MLipo with light (L) irradiation or nano‐Pt alone in 4T1 cells and tumor spheroids. A) The cells were incubated with liposomes for 4 h, and irradiated with 690 nm laser (100 mW cm^−2^) for 1 min. Cell viabilities were examined using CCK‐8 after additional 24 h incubation in fresh medium. B) Cell viability after treatment with free or released nano‐Pt for 48 h. The released nano‐Pt was obtained by light irradiation on the nano‐Pt/VP@MLipo for 1 min. C) Cell apoptosis induced by liposomes plus light irradiation was examined via flow cytometry. The cells were stained with Annexin V‐FITC and PI. D) 4T1 cells treated with liposomes plus irradiation were stained with calcein‐AM and PI for viable (green) and dead (red) cells imaging, respectively. E) Cytotoxicity of liposomes with/without light irradiation in 4T1 tumor spheroids. F) Cytotoxicity of intact liposomes (containing 50 µg mL^−1^ Pt) or those previously treated by light irradiation in 4T tumor spheroids. Low (50 × 10^−9^ m VP, 0.715 µg mL^−1^) and high (200 × 10^−9^ m VP, 2.86 µg mL^−1^ Pt) concentrations of liposomes were used in (A), (C), and (D), and high concentration in (E). Data are presented as mean ± s.d. (*n* = 3). *p < 0.05, ***p < 0.01, and ***p < 0.001.

We then explored the effect of nano‐Pt on the PDT‐mediated cytotoxicity. VP@MLipo and nano‐Pt/VP@MLipo without light irradiation were nontoxic to the tumor cells under the tested condition. Light irradiation alone was also nontoxic (Figure S14, Supporting Information). In contrast, light irradiation‐induced PDT significantly inhibited tumor cell viability, and this effect was further enhanced in the presence of nano‐Pt. Consistent results and observation were obtained in the CCK‐8 test, calcein‐AM/PI dual‐staining assay, and cell apoptosis analysis using flow cytometry (**Figure** **3**A,C,D). Moreover, compared with low‐concentration liposomes (50 × 10^−9^ m VP, 0.715 µg mL^−1^ Pt), photodynamic cytotoxicity using high liposome concentration (200 × 10^−9^ m VP, 2.86 µg mL^−1^ Pt) is much stronger, exhibiting a dose‐dependent manner. Specifically, in 4T1 tumor spheroid model cultured in the ultralow attachment plate as described (Figure S15, Supporting Information),^[^ ^31^ ^]^ PDT in the presence of nano‐Pt induced the death of most tumor cells compared to the partial killing in the treatment without nano‐Pt (Figure 3E).

It is noted that nano‐Pt itself, like other platinum drugs, is cytotoxic.^[^ ^14^ ^]^ However, this effect may be mixed with the PDT effect in the above tests, or due to the limited nano‐Pt exposure to the cells after only 4 h incubation. We then directly evaluated the toxicity of free nano‐Pt and light‐triggered released nano‐Pt on 4T1 cells. It showed that both of them had comparable and strong dose‐dependent cytotoxicity (Figure 3B). We further investigated the toxicity of nano‐Pt in the 4T1 tumor spheroid model. Nano‐Pt/VP@MLipo pretreated with light (releasing nano‐Pt) caused a significant number of tumor cell deaths (Figure 3F), which may be related to the ultrasmall size (3–5 nm) of nano‐Pt, compared to the platinum particle‐loaded liposomes (120 nm), for improved deep penetration and thus access to the cells in tumor spheroid.

### 2.4 Penetration of Nano‐Pt in Agarose Matrix and Tumor Spheroids

**Figure 4:**
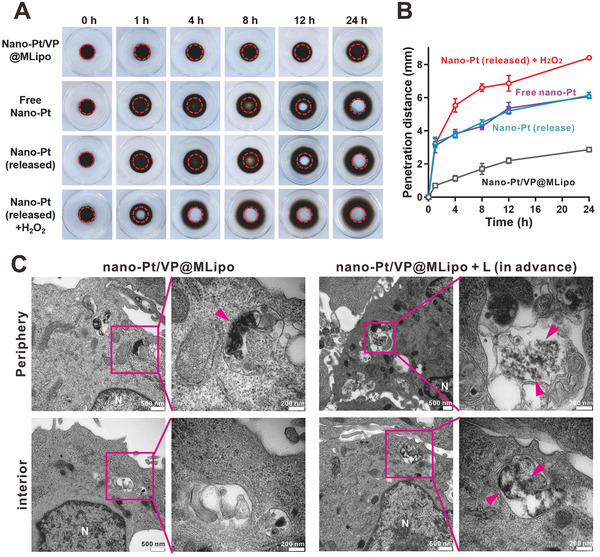
Nano‐Pt penetration in agarose matrix and 4T1 tumor spheroids. A) Penetration of free nano‐Pt and the ones loaded in the liposomes (nano‐Pt/VP@MLipo) in agarose matrix. The Pt concentration used was 500 µg mL^−1^. Representative photographs at different time points were shown. The hole of the gel was indicated with a red dotty line. B) Quantified penetration distance with the time. C) Tumor spheroids were incubated with the intact nano‐Pt/VP@MLipo (50 µg mL^−1^ Pt) or those pretreated with light irradiation (690 nm, 100 mW cm^–2^, 30 s) for 4 h. Both periphery and interior region of the spheroids was examined for nano‐Pt (indicated with pink arrows) and photographed under TEM. Data are presented as mean ± s.d. (*n* = 3).

The penetration capacity of nano‐Pt was characterized using agarose matrix model according to the literature.^[^ ^32^ ^]^ The dark brown nano‐Pt in the hole (≈12 mm diameter) of the gel gradually spread around in a circle over time (**Figure** **4**A). The penetration distance of free nano‐Pt and nano‐Pt released by light irradiation was almost equal. After 24 h, the penetration distance was 6.1 mm, which was 2.1 times further than that (2.9 mm) in the liposomes (nano‐Pt/VP@MLipo). Interestingly, with the involvement of H_2_O_2_, the penetration rate of nano‐Pt (released) was accelerated, reaching 8.4 mm in 24 h (Figure 4B). This suggested that O_2_ generated from H_2_O_2_ decomposition may increase the mobility of nano‐Pt, which could be favorable for their penetration in tumor tissues. Similarly, the self‐propelled approaches by converting chemically powered energy sources into gas bubbles‐driven movement represent an emerging research area of medical nano‐ or microrobots.^[^ ^33^ ^]^

Penetration tests were also investigated in 4T1 tumor spheroids. After 4 h incubation, the spheroids were cut into 90 nm ultrathin sections for detection using TEM. Nano‐Pt loaded in liposomes (nano‐Pt/VP@MLipo) was internalized in the lysosomes only at the periphery of the tumor spheroids (Figure 4C; Figure S16, Supporting Information). In contrast, for the liposomes pretreated with light, nano‐Pt appeared not only in the periphery but also in the interior region of the tumor spheroids. The released nano‐Pt exhibited superior penetrating ability, which would help them play a cytotoxic effect in the deeper tumor parenchyma.

### 2.5 Blood Clearance and Tumor Targeting

**Figure 5:**
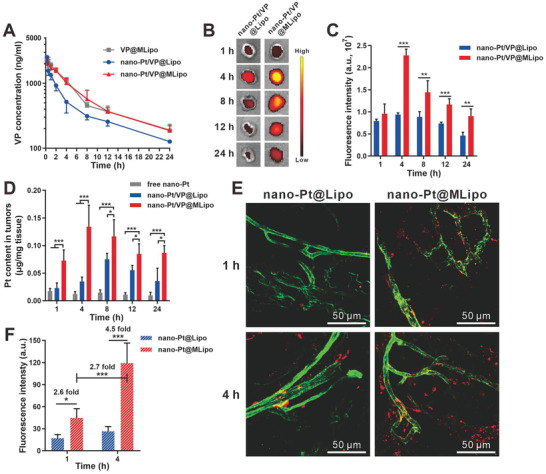
In vivo blood clearance and tumor targeting of nano‐Pt/VP@MLipo. A) VP plasma concentration after i.v. injection of the various liposomes. B) Ex vivo imaging of the tumor at 1, 4, 8, 12, 24 h after liposome injection. VP fluorescence was imaged. C) Quantified fluorescence intensity of the VP‐encapsulated liposomes in tumors in panel (B). D) Pt contents in tumors at different time points after liposome injection. E) Time‐dependent micro‐distribution of DiI‐labeled liposomes (red) in tumors. Tumor vessels (green) were stained with FITC‐labeled lectin. F) Quantitative assay of the liposome fluorescence in panel (E). *n* = 5 in (A). *n* = 4 in (C) and (D). *n* = 3 in (F). Values are presented as mean ± s.d. *p < 0.05, **p < 0.01, and ***p < 0.001.

Long blood circulation is a key property for liposomes to efficiently target tumor sites. The inclusion of M*φ* CM in liposomes conferred a biomimetic feature, as verified in the assay of liposome surface proteins (Figure 1F,G), which would help evade mononuclear phagocytic system (MPS) and extend circulation time of the cargos. VP plasma concentration over the time in healthy BALB/c mice was measured for pharmacokinetic parameter determination. Both nano‐Pt/VP@MLipo (*T*_1/2_, 12.3 h) and VP@MLipo (*T*_1/2_, 12.6 h) had a 1.6 fold longer VP half‐life compared to that (*T*_1/2_, 7.8 h) of conventional liposomes (nano‐Pt/VP@Lipo) without M*φ* CM modification (**Figure** **5**A; Table S2, Supporting Information). Accordingly, nano‐Pt/VP@MLipo and VP@MLipo earned superior AUC_0‐24_ (area under the curve, 17 600.86 and 16 690.12 h ng mL^−1^) and MRT_0‐24_ (mean residence time, 13.96 and 14.25 h), respectively, compared to those (12 543.67 h ng mL^−1^, 10.34 h) of nano‐Pt/VP@Lipo. The Pt concentration in blood was also examined by ICP‐OES (Figure S17, Supporting Information). Due to the limitation of the detection method, Pt content cannot be detected after 4 h. It still showed that Pt content in blood at 2 h and 4 h from nano‐Pt/VP@MLipo was higher than those from nano‐Pt/VP@Lipo, consistent with the observation as for the comparison of VP levels (Figure 5A). Here, we did not directly identify the pharmacokinetics of the nanocarrier. The incorporation of tritiated cholesterol is a smart method to investigate the liposome pharmacokinetics,^[^ ^28^ ^]^ which is worth to be investigated further.

The influence of protein corona on the in vivo fate of nanocarriers is intriguing and recently attracting a lot of attention.^[^ ^34^ ^]^ After 4 h incubation with mouse plasma at 37 ℃, the conventional liposomes (nano‐Pt/VP@Lipo) dominantly adsorbed albumin (MW 66 KDa). However, this was not obviously observed in nano‐Pt/VP@MLipo (Figure S18, Supporting Information), suggesting the property of resisting protein corona. As for nano‐Pt/VP@MLipo, the detected protein bands may, in a considerable portion, come from M*φ* membrane protein due to the M*φ* CM‐hybridized membrane structure.

We explored the time‐dependent distribution in 4T1 tumors through ex vivo imaging of the excised tumors (Figure 5B). The 2.4, 1.6, 1.6, and 2.0 fold increased VP fluorescence in tumors was obtained for nano‐Pt/VP@MLipo compared to that of nano‐Pt/VP@Lipo at 4, 8, 12, and 24 h, respectively (Figure 5C). The peak fluorescence signal was obtained at 4 h, indicating an optimal time point for light irradiation. The improved tumor targeting was accompanied with the significantly decreased distribution in MPS organs (33.4%, 39.3%, and 26.6% decrease in liver, lung, and kidney, respectively) (Figure S19, Supporting Information). Such MPS escape behavior of M*φ* CM‐integrated liposomes are similar to those of “leukosomes,” proteolipid vesicles obtained by incorporating proteins of leukocyte plasma membrane into lipid nanoparticles.^[^ ^17^ ^]^

The nano‐Pt contents in tumors were also examined through ICP‐OES (Figure 5D). At all time points, nano‐Pt from nano‐Pt/VP@MLipo accumulated more in tumor sites compared to the nano‐Pt loaded conventional liposomes. As for free nano‐Pt, they distributed less (below 0.02 µg mg^−1^ tissue) than the two liposome formulations, probably due to their ultrasmall size leading to renal elimination.^[^ ^35^ ^]^

The proteins (such as LFA‐1 and Mac‐1) from M*φ* CM are believed to recognize inflammatory vessels such tumor endothelium.^[^ ^18^ ^]^ We explored the microdistribution of liposomes in tumors under the Multiphoton Laser Scanning Microscope (Olympus FVMPE‐RS) (Figure 5E,F). 1 h after injection, DiI‐labeled nano‐Pt@MLipo (red) dominantly colocalized with the CD31 (green) expressed tumor vessels, exhibiting a tumor vessel targeting property. At 4 h, the liposomes accumulated more and spread widely in the tumor parenchyma while some were still stuck on tumor endothelium. In contrast, conventional liposomes (DiI‐labeled nano‐Pt@Lipo) lacked the ability to target tumor blood vessels, and accumulated less at the tumor site during the same period. The superior targeting to tumor endothelial cells of M*φ* CM‐included liposomes were also verified in vitro using HUVEC as the in vitro model. Nano‐Pt/VP@MLipo adhered more to the HUVEC cells especially when they were stimulated by TNF‐*α* for mimicking the inflamed tumor endothelium (Figure S20, Supporting Information).

### 2.6 In Vivo Antitumor Therapy

**Figure 6:**
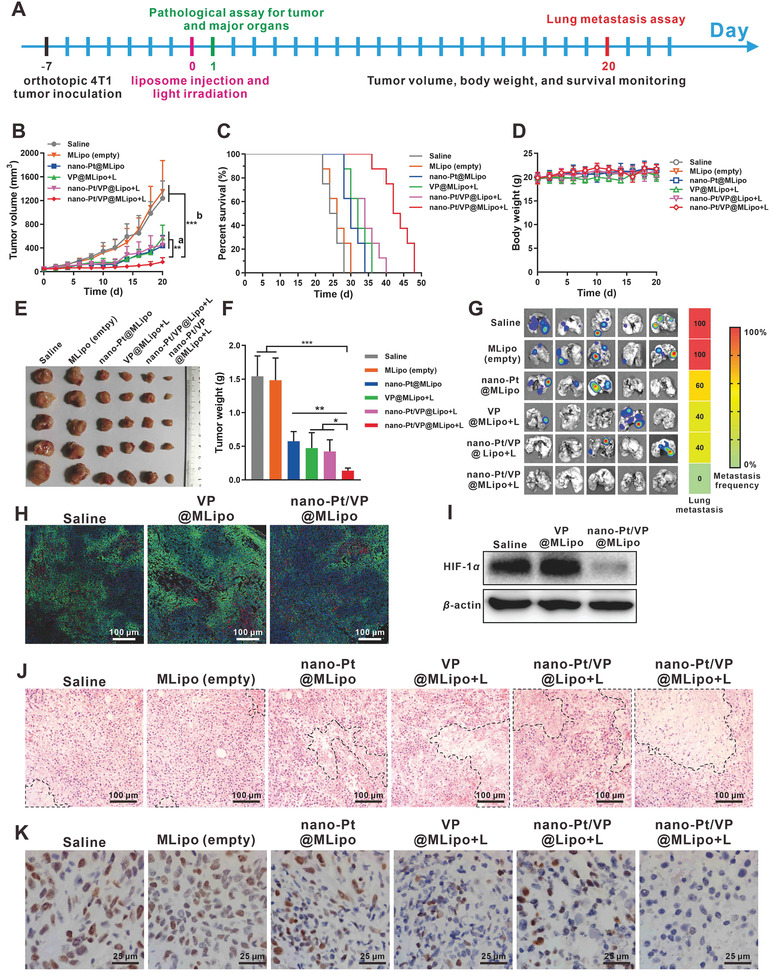
Antitumor therapy in vivo. A) Treatment program. B) Tumor volume growth curves till day 20. a) Nano‐Pt/VP@MLipo+L versus nano‐Pt/VP@Lipo+L or VP@MLipo+L or nano‐Pt@MLipo. b) Nano‐Pt/VP@MLipo+L versus MLipo (empty) or saline. C) Mouse survival curves. D) Mouse body weight. E) Photographs of the tumors harvested on day 20. F) Tumor weight on day 20. G) Ex vivo bioluminescence imaging of the lung metastasis. Heat map summarizes the metastasis frequencies. H) Pimonidazole positive hypoxic areas in 4T1 tumors. I) Western blot assay of HIF‐1*α* expression in 4T1 tumors 4 h after i.v. injection of liposomes. J) H&E staining of the tumor paraffin section. The necrotic area is outline with dotted line. K) The brown nuclei of proliferative tumor cells were stained with anti‐PCNA antibody. *n* = 8 in (B), (C), and (D). *n* = 5 in (F). *n* = 3 in (I). Values are presented as mean ± s.d. *p < 0.05, **p < 0.01, and ***p < 0.001.

Based on the extended circulation time and enhanced tumor targeting, we then explored the antitumor potency of the liposomes in the orthotopic 4T1 breast tumor mouse model. The therapy program was performed with the schedule in **Figure** **6**A. When the tumor grew to ≈50 mm^3^ (7 days after cell inoculation), animals were randomly allocated into 6 groups and i.v. dosed with various liposomes with 0.25 mg kg^−1^ VP and 5 mg kg^−1^ Pt when involved. The tumors were irradiated (690 nm, 100 mW cm^−2^, 10 min) 4 h after injection, and then the tumor growth was monitored.

Nano‐Pt/VP@Lipo + L (light), VP@MLipo +L, and nano‐Pt@MLipo alone obviously hindered tumor growth, compared to the rapid growth in the groups of saline and MLipo (empty). The strongest inhibitory effect was earned in the group of Nano‐Pt/VP@MLipo +L, showing ≈90% tumor inhibition in the same period (Figure 6B,E,F; Figure S21, Supporting Information). Accordingly, treatment with nano‐Pt/VP@MLipo +L extended the mice survival (median survival 43 days) compared to other treatments, including saline (25 days), MLipo (empty) (26 days), nano‐Pt@MLipo (30 days), VP@MLipo +L (32 days), and nano‐Pt/VP@Lipo +L (34 days) (Figure 6C). Nano‐Pt/VP@MLipo +L treatment resulted in the longest increase in survival of mice by 72% (Table S3, Supporting Information).

Lung metastasis was examined using bioluminescence imaging, as the metastasis in lung is common for 4T1 tumor in mice.^[^ ^36^ ^]^ Compared to the observation that 2–5 mice in saline or other control groups had metastasis in lung, no lung metastasis occurred to the mice treated with nano‐Pt/VP@MLipo+L (Figure 6G). This inhibition of lung metastasis may be related to the enhanced suppression of the orthotopic tumor. The treatment with nano‐Pt/VP@MLipo+L did not introduce loss of body weight, indicating low acute toxicity (Figure 6D).

The hypoxic status of tumors was also evaluated. Regions with comparable tumor microvessel density (CD31^+^ staining) were adopted for analysis (Figure S22, Supporting Information). It showed nano‐Pt‐involved treatment dramatically decreased the hypoxic region (pimonidazole staining) by 78.4%, compared to the therapy without nano‐Pt (Figure 6H; Figure S23, Supporting Information). Meanwhile, HIF‐1*α* expression in tumor tissues, which was examined by immunohistochemical assay (Figure S24, Supporting Information) and western blot (Figure 6I), was dramatically reduced, reflecting the significantly reduced hypoxia, which would potentially enhance VP‐mediated PDT.

Histopathological examination showed that nano‐Pt/VP@MLipo+L induced more necrosis (Figure 6J; Figure S25A, Supporting Information) and fewer PCNA positive proliferative cells (Figure 6K; Figure S25B, Supporting Information) in tumors compared with other controls. Pathological examination (hematoxylin and eosin staining) of major organs in all groups exhibited no obvious histological toxicity together with the saline group (Figure S26, Supporting Information). Blood analysis was further carried out to examine the treatment‐associated toxicity. Typical parameters relative to the function of liver and kidney and hematological toxicity were examined (Figure S27, Supporting Information). No significant difference in the parameters of nano‐Pt/VP@MLipo+L group, when compared to the saline group, suggesting the good tolerance of the therapy at the dose given.

We further performed dose escalation study to explore the maximum tolerated dose. The 2‐, 5‐, and 10‐fold elevated doses of nano‐Pt/VP@MLipo containing nano‐Pt (10, 25, 50 mg kg^−1^) and corresponding VP (0.5, 1.25, and 2.5 mg kg^−1^), respectively, were tested. It showed the numbers of white blood cells and platelets in blood dramatically declined under the highest dose (nano‐Pt 50 mg kg^−1^, VP 2.5 mg kg^−1^) (Figure S28, Supporting Information). However, no mice died in 24 h after i.v. injection before they were sacrificed for blood sampling, exhibiting relatively good tolerance of the liposomal formulation.

## 3 Conclusion

In summary, this study presents the first attempt to exert the multifunctional antitumor effects of nano‐Pt, based on a liposome‐based delivery system engineered through reverse phase evaporation and M*φ* cell membrane camouflage. The biomimetic nano‐Pt/VP@MLipo effectively targeted the tumor sites, where oxygen produced by nano‐Pt catalyzation enhanced the VP‐mediated PDT. PDT in turn permeabilized the liposome membrane for efficient nano‐Pt release. These ultrasmall particles (3–5 nm) achieved penetration in deeper tumor tissue, which was also facilitated by the generated oxygen gas, for enhanced chemotherapy. This integrated chemophototherapy with nano‐Pt shows potent antitumor effect in overcoming 4T1 tumor and its lung metastasis and extending the mouse survival without overt toxicity.

## 4 Experimental Section

##### Materials, Cell, and Animal

Chloroplatinic acid hexahydrate (H_2_PtCl_6_), sodium borohydride (NaBH_4_), VP, DiO, DiI, DiR, and DPBF were purchased from Sigma‐Aldrich (St. Louis, MO). 1,2‐Dipalmitoyl‐*sn*‐glycero‐3‐phosphocholine (DPPC), 1,2‐dioleoyl‐*sn*‐glycero‐3‐phosphocholine (DOPC), 1,2‐distearoyl‐*sn*‐glycero‐3‐phosphocholine (DSPC), and cholesterol were purchased from Avanti Polar Lipids (Alabama, USA). Tris(4,7‐diphenylL‐1,10‐phenanthroline)ruthenium(II)dichloride ([Ru(dpp)_3_]Cl_2_) was purchased from Alfa Aesar (Shanghai, China). 2′,7′‐dichlorofluorescein diacetate (DCFH‐DA) was obtained from R&D systems (Shanghai, China). FITC labeled rabbit antimouse LAF‐1, Mac‐1, CD45, CD47, and goat antimouse IgG were purchased from BD Biosciences (Shanghai, China). Recombinant Human TNF‐*α* was obtained from Peprotech (Suzhou, China). Cell counting kit‐8 (CCK‐8) was purchased from Dojindo Laboratories (Kumamoto, Japan). Annexin V‐FITC, propidium iodide (PI), Dulbecco's modified Eagle's medium (DMEM), fetal bovine serum (FBS), penicillin, streptomycin, and trypsin were purchased from ThermoFisher Scientific (Shanghai, China). Other reagents were of analytical grade and used without purification.

Mouse 4T1 breast cancer cell line and RAW264.7 macrophage were obtained from ATCC (Manassas, VA). Primary human umbilical vein endothelial cells (HUVEC) were from Lifeline Cell Technology (Frederick, MD). The luciferase transfected 4T1 cells (4T1‐luc) were established by Shanghai Model Organisms Center (China). The cells were cultured in DMEM supplemented with 10% FBS, 100 U mL^–1^ penicillin, and 100 µg mL^–1^ streptomycin and in the incubator containing 5% CO_2_ under 37°C.

BALB/c mice (female, age of 6–7 weeks) were obtained from Shanghai Laboratory Animal Center (China). All animals involved tests were approved by the SJTU‐SM IACUC.

##### Preparation and Characterization of Nano‐Pt/VP@MLipo

Pt nanoparticles (nano‐Pt, 3–5 nm) were prepared through reduction of H_2_PtCl_6_ by NaBH_4_ in the presence of folic acid as described.^[^ ^23^ ^]^ The solution color changed from yellow to brown black, indicating the generation of nano‐Pt. The product was purified through ultrafiltration (Amicon Ultra‐15 Centrifugal Filter Units, 30 000 MWCO) and washed with PBS for 3 times.

Nano‐Pt/VP@MLipo was prepared through reverse phase evaporation technique^[^ ^16^ ^]^ and a follow‐up freeze–thaw hybridization with RAW264.7 cell membrane (CM) vesicles. Briefly, DPPC, DOPC, DSPC, and cholesterol were separately dissolved in chloroform, and then mixed at the mass 3.6, 2.4, 0.79, and 0.38 mg, respectively. The corresponding molar ratio of the lipid was 5:3:1:1, and the typical total chloroform volume is 800 µL. Then verteporfin (108 µg) in 50 µL chloroform and 3 mL diethyl ether were added to form the oil phase. Then, nano‐Pt (4 mg) in 1 mL PBS as aqueous phase was added and then 10 min water bath sonication was introduced at a frequency of 42 kHz and a power of 100 W. The resulted water‐in‐oil emulsion was then transferred to a round‐bottom flask, and organic solvents were evaporated using a rotary evaporator at 30 °C till the gel phase disappeared. Then, 4 mL PBS containing the M*φ* CM with 0.36 mg membrane protein was added. The mixture was then subjected to 5 freeze–thaw cycles (5 min per cycle) between 0 and 45 °C for the hybridization of lipid and M*φ* CM. Then the solution was extruded through a 200 nm diameter polycarbonate membrane using a miniextruder (Avanti Polar Lipids). The resulted nano‐Pt/VP@MLipo were obtained through centrifugation (12 000 rpm, 10 min). Nano‐Pt/VP@Lipo and empty MLipo were also prepared as controls using the same method while without the addition of corresponding components (M*φ* CM, nano‐Pt, or VP).

The macrophage CM vesicles were prepared as previously described. ^[^ ^37^ ^]^ Briefly, 1 × 10^8^ macrophages were dispersed in 20 mL hypotonic solution (1 mmol L^−1^ NaHCO_3_, 0.2 mmol L^−1^ EDTA, 1 mmol L^−1^ PMSF) at 4 °C over night. Then, the cell solution was homogenized using Precellys (Bertin Technologies). The homogenized solution was centrifuged at 4500 × *g* for 5 min at 4 °C and the supernatant was then centrifuged at 15 000 × *g* for 30 min at 4 °C. The resulting CM pellet was resuspended and sonicated using a bath sonicator (Fisher Scientific) at a frequency of 42 kHz and power of 100 W for 4 min for following use.

The hydrodynamic diameter and zeta potential of the liposomes were examined through DLS using ZetaSizer Nano ZS instrument (Malvern, UK). The nanoparticle shape and structure were characterized using TEM (FEI Talos F200X) and Cryo‐TEM (Talos F200C G2). Light irradiation‐induced nano‐Pt leakage and size change were examined using TEM and DLS.

The content and encapsulation efficiencies of the liposomes were measured by ICP‐OES (iCAP6300, Thermo Scientific) for nano‐Pt and fluorescence assay (Ex 405 nm, Em 690 nm) for VP after proper pretreatment of the liposomes or liposome‐contained tissues.

For membrane protein assay, M*φ* CM vesicles and the hybridized liposomes were treated with RIPA lysis buffer and the membrane proteins in the supernatant were obtained by centrifugation (12 000 rpm, 20 min). Membrane proteins (25 µg) quantified by BCA Protein Assay Kit were subjected to SDS‐PAGE and Coomassie Blue staining.

##### Identification of Membrane Hybridization

FRET test was used for membrane hybridization assay. Briefly, liposomes were labeled with DiO (FRET donor) and DiI (FRET acceptor) at equal concentration (5 × 10^−6^ m). Then, M*φ* CM vesicles were added for repeated freeze–thaw treatment. The fluorescence intensities of DiO (Ex 450 nm, Em 500 nm) and FRET signal (Ex 450 nm, Em 565 nm) were detected using a SpectraMAX M2 microplate reader (Molecular Devices). The fusion of M*φ* CM with liposomes will increase the distance between the two dyes and lead to a recovery of the DiO fluorescence emission at 500 nm and accompanied decreased FRET intensity at 565 nm.

Membrane hybridization was also observed using confocal microscopy. For this assay, DiO‐labeled M*φ* CM and DiR‐labeled liposomes were extruded without previous freeze–thaw or subjected to both freeze–thaw and follow‐up extrusion. Then, the samples were dispersed in glycerol for decreased mobility ^[^ ^38^ ^]^ and the colocalization of the two dyes were observed under CLSM (Leica TCS SP8, Germany; DiO: Ex/Em = 488 nm/501 nm; DiR: Ex/Em = 633 nm/750 nm).

##### Assay of the M*φ*‐Associated Proteins and Their Orientation on Liposome Surface

Macrophage membrane proteins and their correct orientation on the surface of nano‐Pt/VP@MLipo were verified using flow cytometry. nano‐Pt/VP@MLipo was diluted in FACS buffer solution (PBS, 1% BSA) to a VP concentration of 10 × 10^−6^ m. Then, FITC‐labeled anti‐LFA‐1, anti‐Mac‐1, anti‐CD45, and anti‐CD47 (2.5 µg mL^−1^) for protein's extracellular domain binding were added separately for following 30 min incubation at room temperature. Free antibody was removed by centrifugation. The antibody fluorescence intensity on the liposome surface were detected using the flow cytometer. Nano‐Pt/VP@Lipo and the incubation of FITC‐labeled goat antimouse IgG with the liposomes were included as control.

##### Catalase‐Like Activity of Nano‐Pt/VP@MLipo

Catalase‐like activity of nano‐Pt/VP@MLipo was detected by measuring O_2_ generation using a fiber‐optic oxygen sensor (Oxylite, Oxford Optronix, UK).^[^ ^39^ ^]^ Nano‐Pt/VP@MLipo with 1 or 10 µg mL^−1^ Pt were dispersed in water containing 100 × 10^−6^ m hydrogen peroxide (H_2_O_2_) and the oxygen levels were recorded over time. The dissolved O_2_ in hydrogen peroxide solution was previously removed by introducing N_2_ for 20 min and the experiment was carried out in hypoxia in the Captair Pyramid (Erlab, Beijing, China) with pO_2_ below 5 mmHg.

The O_2_ generation through catalyzing high level of intracellular H_2_O_2_ in tumor cells was investigated by confocal fluorescence imaging with an O_2_ sensing probe, [(Ru(dpp)_3_)]Cl_2_. The fluorescence of this agent can be strongly quenched by O_2_.^[^ ^6^ ^]^ 4T1 cells were cultured at a density of 5 × 10^3^ cells per well in 96‐well plate and under hypoxia (1% O_2_, 5% CO_2_ balanced with N_2_) for 24 h. Then, the cells were incubated with 5 × 10^−6^ m [Ru(dpp)_3_]Cl_2_ for 4 h and with nano‐Pt/VP@MLipo (200 × 10^−9^ m VP, 2.86 µg mL^−1^ Pt) for another 4 h. The fluorescent signal of [Ru(dpp)_3_]Cl_2_ (Ex 488 nm, Em 610 nm) in cells was then observed and photographed under CLSM.

##### Reactive Oxygen Species (^1^O_2_) Generation Assay

DPBF was used for the detection of ^1^O_2_ generated by nano‐Pt/VP@MLipo in the presence of H_2_O_2_ upon 690 nm laser irradiation. DPBF could be oxidized by ROS and result in a decrease in its absorption at 410 nm.^[^ ^40^ ^]^ In a typical process, 5 µL DPBF (1 mg mL^−1^ in DMSO) was added to 200 µL PBS solution containing nano‐Pt/VP@MLipo or VP@MLipo (VP 200 × 10^−9^ m, Pt 2.86 µg mL^−1^) with or without H_2_O_2_ (100 × 10^−6^ m). Then the mixture was irradiated under 690 nm laser (100 mW cm^−2^) for various time periods. DPBF absorption at 410 nm was measured to examine the ^1^O_2_ generation. The test was performed under hypoxia (pO_2_ < 5 mmHg) in the Captair Pyramid (Erlab, Beijing, China).

Intracellular ROS production was examined using DCFH‐DA as described.^[^ ^40^ ^]^ DCFH‐DA could enter into living cells and then be deacetylated by intracellular esterase to form DCFH, which could be further oxidized by ROS to generate fluorescent 2.7‐dichlorofluorescein (DCF). Thus, the fluorescence intensity of DCF reflects the ROS level within the cells. 4T1 cells in 96 well‐plate were cultured in hypoxic environment (1% O_2_, 5% CO_2_ balanced with N_2_). The cells were then incubated with nano‐Pt/VP@MLipo (VP 200 × 10^−9^ m, Pt 2.86 µg mL^−1^) for 4 h. Then, the cells were washed with PBS, and incubated with serum‐free medium containing 1 × 10^−6^ m DCFH‐DA for 30 min. The cells were then irradiated with 690 nm laser (100 mW cm^−2^) for 1 min and the intracellular fluorescence was observed and photographed in the IncuCyte Live‐Cell Analysis System (Essen BioScience).

##### Expression and Intracellular Localization of HIF‐1*α* Protein

HIF‐1*α* expression was detected using western blot. For in vitro assay, 1 × 10^5^ 4T1 cells were seeded in 6‐well plates and incubated for 24 h under hypoxia (1% O_2_, 5% CO_2_ balanced with N_2_). Then, the cells were incubated with nano‐Pt/VP@MLipo or VP@MLipo (VP 200 × 10^−9^ m, Pt 2.86 µg mL^−1^) for additional 1 and 4 h, respectively. The cells that were always cultured under hypoxia or normoxia (5% CO_2_) conditions or hypoxia followed by 1 or 4 h normoxia were included as controls. For in vivo assay, 4 h after the liposome injection (containing VP 0.25 mg kg^−1^, Pt 5 mg kg^−1^), 4T1 tumors from the mice were excised.

Proteins from 4T1 cells or homogenized 4T1 tumors were extracted using RIPA Lysis Buffer with Complete Protease Inhibitor Cocktail Tablets (Roche). For each sample, 40 mg of protein determined by BCA Protein Assay Kit (Thermo Scientific, Rockford, IL) were applied to 10% SDS‐PAGE, electrically transferred to Immobilon‐P membranes, and incubated with rabbit anti‐HIF‐1*α* antibody (1:1000, abcam) and horseradish peroxidase‐conjugated goat antirabbit antibody for color development as previously described.^[^ ^39^ ^]^

HIF‐1*α* expression and intracellular localization were also observed using immunofluorescence assay. 5 × 10^4^ 4T1 cells were cultured in 24‐well plates on glass coverslips. After various treatments in hypoxia or normoxia in the presence of nano‐Pt/VP@MLipo like the western blot assay, the cells were stained with rabbit anti‐HIF‐1*α* antibody (1:1000, abcam) overnight at 4 °C and then incubated with Alexa Fluor 647‐labeled goat anti‐rabbit IgG secondary antibody (1:1000) for 1 h at room temperature. The F‐actin was labeled with FITC‐labeled phalloidin (Ex 488 nm, Em 525 nm) to outline the cells. The nuclei were stained with the Hoechst 33342 (Ex 405 nm, Em 450–460 nm). The fluorescence intensity of the antibody‐labeled HIF‐1*α* (Ex 633 nm, Em 650 nm) and its localization were observed using Leica TCS‐SP8 CLSM.

##### Cell Viability Assay

4T1 cells (5 ×10^3^ cell per well) were cultured in 96‐well plates under hypoxia. The cells were incubated with nano‐Pt/VP@MLipo for 4 h. Then, the cells were incubated in fresh medium, and irradiated with 690 nm laser (100 mW cm^−2^) for 1 min. After 24 h, cell viability was assayed using Cell Counting Kit‐8 according to the manufacturer's instructions. Nano‐Pt/VP@MLipo alone without laser and VP@MLipo with/without laser were included as controls.

Cytotoxicity after the same treatments were also examined using flow cytometry (Cytomics FC500, Beckman Coulter) after FITC‐Annexin V and PI staining or qualitatively examined by calcein‐AM/PI dual‐staining assay in 4T1 cells or the tumor spheroid model.

The toxicity of nano‐Pt, both free and light‐triggered released ones from the liposomes, to 4T1 cells or tumor spheroids were also evaluated. The cells were incubated with nano‐Pt for 48 h, and cell viability was examined using CCK‐8 or calcein‐AM/PI dual‐staining test.

##### 4T1 Tumor Spheroid and Penetration Assay

The 3D multicellular spheroids were cultured according to the described method.^[^ ^31^ ^]^ Briefly, 4T1 cells were seeded at a density of 7 × 10^3^ per well in ultralow attachment, round bottom 96‐well plates. Then, the plates were centrifuged at 1000 × *g* for 10 min at 4 °C. The cells were then allowed to aggregate on the bottom of the plate and form tumor spheroid after 48 h culture under hypoxia (1% O_2_, 5% CO_2_ balanced with N_2_).

To investigate the penetration of nano‐Pt in 4T1 tumor spheroids, intact nano‐Pt/VP@MLipo (Pt 50 µg mL^−1^) or the liposomes previously treated with 690 nm light irradiation (100 mW cm^–2^, 1 min) were added to the tumor spheroid‐contained 96‐well plate for 4 h incubation. Then, the tumor spheroids were fixed in Karnovsky's fixative, and embedded in epoxy resin and propylene oxide. The spheroids were then cut into ultrathin sections with 90 nm thickness, and stained with lead citrate for TEM (PHILIP CM‐120) observation.^[^ ^41^ ^]^

##### Nano‐Pt Penetration in Agarose Matrix Model

The penetration behavior of nano‐Pt was evaluated using agarose matrix according to the literature.^[^ ^32^ ^]^ Agarose gels were prepared by heating a suspension of agarose in PBS (0.75 wt%) in a microwave oven. Then, 3 mL clear agarose solution was poured into the lid of Corning cell culture dish (35 mm×10 mm). When the gel with uniformed thickness and no bubbles and fissures was formed at room temperature, a hole was punched using a glass tube with a diameter of 12 mm in the middle of the gel. The gels were then soaked in PBS for 12 h at 37 °C in the incubator for gel saturation. For penetration test, nano‐Pt/VP@MLipo, free nano‐Pt, or released nano‐Pt from nano‐Pt/VP@MLipo in 150 µL PBS were added to the gel hole. To examine the influence of H_2_O_2_ on nano‐Pt penetration, released nano‐Pt in 150 µL H_2_O_2_ (100 × 10^−6^ m) was added to the hole of the gel presoaked in H_2_O_2_ (100 × 10^−6^ m). Pt concentration used was 500 µg mL^−1^. The gels were placed at 37 °C in the humidified incubator throughout the test. After 1, 4, 8, 12, and 24 h, the gels were photographed using digital camera, and the boundary and penetration distance of the nano‐Pt were identified and estimated using ImageJ software.

##### Blood Clearance Kinetics

Female BALB/c mice (*n* = 5) were intravenously injected via tail vein with nano‐Pt/VP@MLipo, nano‐Pt/VP@Lipo, and VP@MLipo (VP 0.25 mg kg^−1^, Pt 5 mg kg^−1^). At predetermined time points of 0.25, 0.5, 1, 2, 4, 8, 12, and 24 h post injection, 50 µL blood was sampled from orbital vein for VP and Pt quantification. For VP assay, blood was centrifuged at 1500 × *g* for 15 min. 20 µL plasma was diluted in 130 µL the mixture of methanol and tetrahydrofuran (1:3)^[^ ^42^ ^]^ to destroy the liposome structure. Then, the samples were dried using the SpeedVac vacuum concentrator (Thermo Scientific), dispersed in DMSO. VP content was measured by fluorescence detection (Ex 405 nm, Em 690 nm). The VP concentration versus time curve was then obtained and the half‐life in blood was estimated using the WinNonlin software (Version 6.1 Pharsight, CA) using the noncompartmental model. Pt content in whole blood was quantified using ICP‐OES.

##### Protein Corona Assay

Protein corona was assayed according to the literature.^[^ ^43^ ^]^ Liposomes (nano‐Pt/VP@Lipo, nano‐Pt/VP@MLipo) with VP 0.025 mg mL^−1^ and Pt 0.5 mg mL^−1^ were incubated with 100 µL mouse plasma at 37 °C for 4 h. The mixture was then centrifuged at 12 000 rpm for 20 min, and the pellet was rinsed with cold PBS three times. The proteins adsorbed on the liposomes were detected using SDS‐PAGE. 1 µL mouse plasma was loaded as control.

##### Tumor Targeting and Biodistribution

Orthotopic 4T1 tumor model was established through inoculating 1 × 10^6^ 4T1‐luc cells (in 50 µL PBS) into the right fourth mammary fat pad of female BALB/c mice. When tumor grew to ≈50 mm^3^, nano‐Pt/VP@MLipo or nano‐Pt/VP@Lipo (VP 0.25 mg kg^−1^, Pt 5 mg kg^−1^) was i.v. injected to the mice. After 1, 4, 8, 12, and 24 h, the mice were sacrificed and tumors or major organs (heart, liver, spleen, lung, and kidney) were harvest for ex vivo imaging of VP signal (Ex 650 nm, Em 690 nm) using the IVIS Spectrum CT imaging system (PerkinElmer). The VP fluorescence intensity was quantified and compared. Time‐dependent (1–24 h) Pt contents in tumors after liposome or free nano‐Pt injection were also assayed using ICP‐OES.

##### Time‐Dependent Microdistribution of the Liposomes in Tumors

When tumor grew to ≈50 mm^3^, mice were i.v. injected with DiI‐labeled nano‐Pt@MLipo or nano‐Pt@Lipo (containing DiI 45 µg kg^−1^). Tumor vessels were labeled with FITC‐labeled *Bandeiraea simplicifolia* Lectin (2 mg kg^−1^) via tail vein injection 30 min before the mice were sacrificed. After 1 and 4 h after liposome injection, tumors were harvested and fixed with 4% paraformaldehyde, the fluorescence of DiI (Ex 960 nm, Em 567 nm) and FITC‐labeled Lectin (Ex 960 nm, Em 520 nm) were observed using a Multiphoton Laser Scanning Microscope (Olympus FVMPE‐RS, Japan) to track the liposome distribution with reference to the blood vessels. The liposome fluorescent intensity in tumors was quantified using ImagJ software.

##### Tumor Hypoxia Identification

Pimonidazole, a common hypoxia probe, was used to identify the oxygen level in tumors.^[^ ^44^ ^]^ When tumor grew to ≈50 mm^3^, nano‐Pt/VP@MLipo (VP 0.25 mg kg^−1^, Pt 5 mg kg^−1^) was i.v. injected. After 3 h, pimonidazole (1.5 mg) was i.p. injected to the mice. After additional 1 h, the mice were sacrificed and the tumors were excised and processed for frozen section. For the detection of the pimonidazole adducts, the sections were stained with Hypoxyprobe‐1‐Mab1 (Hypoxyprobe‐1 Kit, MA) and donkey anti‐rabbit IgG H&L (Alexa Fluor 546, Invitrogen) secondary antibody, and observed under CLSM (Ex 561 nm, Em 600 nm). The CD31^+^ vessels were also stained with rat antimouse CD31 antibody (1: 200, BD biosciences) and goat anti‐rat IgG H&L (Alexa Fluor 647, Invitrogen) (Ex 633 nm, Em 650 nm)

##### Antitumor Therapy In Vivo

When 4T1‐luc tumor grew to ≈50 mm^3^, mice were randomly divided into six groups as follows: 1) saline; 2) MLipo (empty); 3) nano‐Pt@MLipo; 4) VP@MLipo+L (light); 5) nano‐Pt/VP@Lipo+L; 6) nano‐Pt/VP@MLipo+L. The dose of VP was 0.25 mg kg^−1^ and Pt was 5 mg kg^−1^ when involved. 4 h after i.v. injection, the tumors were irradiated with laser (690 nm, 100 mW cm^−2^) for 10 min. Tumor volume and body weight were recorded every 2 days. The tumor volume was calculated as follows: *V* = (length) × (width)^2^/2. Survival (*n* = 8 per group) was recorded until tumor volume reached the ethical limit (2000 mm^3^).^[^ ^45^ ^]^ Increase in life span (ILS) was obtained according to the following formula: %ILS = (*T*/*C* − 1) × 100%. *T* and *C* were the median survival time of mice in the treated and saline group, respectively.

24 h after injection, tumors and major organs from 3 mice per group were excised, fixed, and processed for paraffin section. Tumor and organ sections were stained with H&E for histopathological evaluation. Tumor cell proliferation in tumor tissues was identified using PCNA cell proliferation kit. All images were taken by Nikon Eclipse E200 photomicroscope and analyzed using Image‐Pro Plus 6.0 software (Media Cybernetics, Bethesda, MD).

In a separate study, on day 20 when there was a mouse's tumor grew above 2000 mm^3^, 5 mice from each group were sacrificed. The tumors were photographed and weighted, and the mouse lungs were harvested for ex vivo bioluminescent imaging to examine the metastasis as previously described.^[^ ^46^ ^]^

##### Blood Analysis

Healthy female BALB/c mice were intravenously injected with saline, MLipo (empty), nano‐Pt/VP@Lipo, and nano‐Pt/VP@MLipo (VP 0.25 mg kg^−1^, Pt 5 mg kg^−1^), respectively. 24 h later, retro orbital blood collection from 3 mice per group were performed for serum biochemistry assay and complete blood panel analysis, which were conducted in Drug Safety Evaluation Research Center (Shanghai Institute of Materia Medica, Shanghai, China). Blood analysis was also performed in the dose escalation study, in which 2‐, 5‐, and 10‐fold elevated doses of nano‐Pt/VP@MLipo containing nano‐Pt (10, 25, 50 mg kg^−1^) and corresponding VP (0.5, 1.25, and 2.5 mg kg^−1^), respectively, were tested.

##### Statistical Analysis

Statistical analysis was performed using GraphPad Prism 6.0 software (La Jolla, CA). Student's *t*‐test or ANOVA with Tukey's multiple comparison tests were used for the examination of differences between groups, which were considered significant if *p*‐value was below 0.05.

## Conflict of Interest

The authors declare no conflict of interest.

## Supporting information

Supporting Information

## Data Availability

Research data are not shared.
