# Correction to “Biomimetic Liposomal Nanoplatinum for Targeted Cancer Chemophototherapy”

**DOI:** 10.1002/advs.202412806

**Published:** 2025-04-26

Xue‐Liang Liu, Xiao Dong, Si‐Cong Yang, Xing Lai, Hai‐Jun Liu, Yuhao Gao, Hai‐Yi Feng, Mao‐Hua Zhu, Yihang Yuan, Qin Lu, Jonathan F. Lovell, Hong‐Zhuan Chen*, Chao Fang*. Biomimetic Liposomal Nanoplatinum for Targeted Cancer Chemophototherapy. *Adv Sci*, 2021, 8, 2003679.

The image of the saline group (untreated control) in Figure 6J was inadvertently derived from the MLipo (empty) group (a drug‐free control). The corrected “Saline” panel for Figure 6J is presented below.


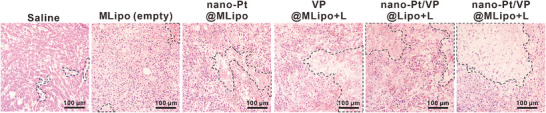


**Figure 6J**. H&E staining of the tumor paraffin section. Necrotic areas are outlined with dotted lines.

The authors apologize for this error and for the inconvenience it may have caused.

