# Supplementary material for: Biomimetic Liposomal Nanoplatinum for Targeted Cancer Chemophototherapy
Source: Adv Sci (Weinh). 2021 Mar 1;8(8):2003679. doi: 10.1002/advs.202003679 (PMC8061387; doi:10.1002/advs.202003679)
Supplement: Supplementary file 1 — Supporting Information [file ADVS-8-2003679-s001.pdf]

© 2021 Wiley-VCH GmbH

ADVANCED SCIENCE
Open Access

Supporting Information

for *Adv. Sci.*, DOI: 10.1002/advs.202003679

Biomimetic Liposomal Nano-Platinum for Targeted Cancer Chemophototherapy

Xue-Liang Liu, Xiao Dong, Si-Cong Yang, Xing Lai, Hai-Jun Liu, Yuhao Gao, Hai-Yi Feng, Mao-Hua Zhu, Yihang Yuan, Qin Lu, Jonathan F. Lovell, Hong-Zhuan Chen and Chao Fang**

Supporting Information

Biomimetic Liposomal Nano-Platinum for Targeted Cancer Chemophototherapy

Xue-Liang Liu, Xiao Dong, Si-Cong Yang, Xing Lai, Hai-Jun Liu, Yuhao Gao, Hai-Yi Feng, Mao-Hua Zhu, Yihang Yuan, Qin Lu, Jonathan F. Lovell, Hong-Zhuan Chen and Chao Fang**

Table S1 Physicochemical characterization of various liposomal formulations and Mφ CM vesicles. Values are expressed as mean ± s.d. (n=3).

Liposomes and Mφ CM vesicles	Particle size (nm)	PDI	Zeta potential (mV)	EE%[e] (Pt)	EE% (VP)	DL%[f] (Pt)	DL% (VP)
Mφ CM vesicles[a]	231.5±12.7	0.230±0.029	-23.4±2.5	-	-	-	-
VP@MLipo[b]	121.6±3.3	0.122±0.014	-14.0±1.1	-	91.2±1.7	-	1.17±0.04
nano-Pt/VP@Lipo[c]	143.1±3.5	0.164±0.013	-11.6±1.2	45.6±1.5	90.5±2.5	17.5±0.4	1.01±0.03
nano-Pt/VP@MLipo[d]	142.9±3.4	0.117±0.016	-16.7±2.7	48.2±0.7	89.5±1.6	15.3±0.4	0.87±0.03

[a] Mφ CM vesicles, macrophage (Mφ) cell membrane (CM) vesicles.
[b] VP@MLipo, VP-encapsulated liposomes hybridized with Mφ CM vesicles.
[c] nano-Pt/VP@Lipo, nano-Pt and VP co-loaded conventional liposomes
[d] nano-Pt/VP@MLipo, nano-Pt and VP co-loaded liposomes hybridized with Mφ CM vesicles.
[e] EE, Encapsulation efficiency.
[f] DL, Drug loading.

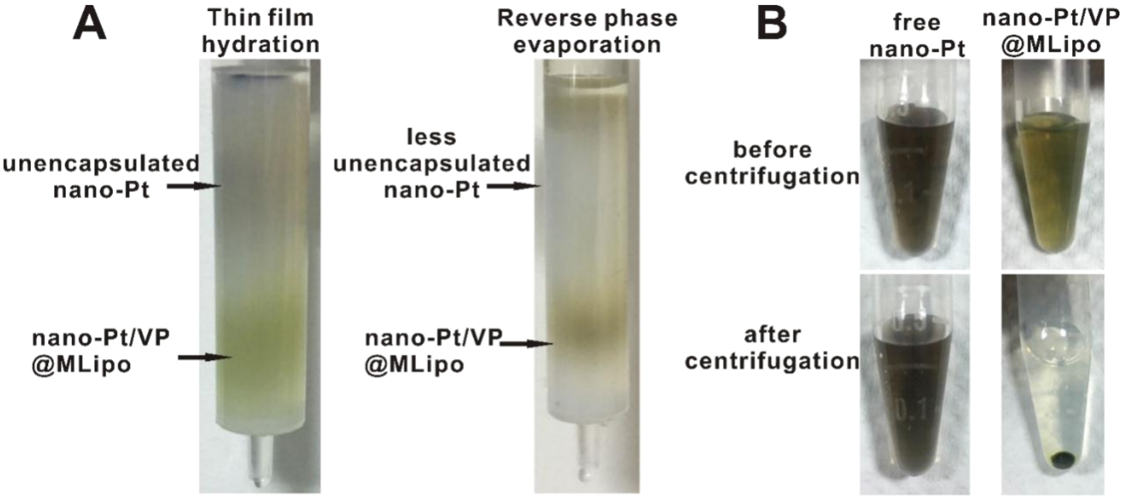

Figure S1. Liposome purification. (**A**) Free un-encapsulated nano-Pt can be separated from the liposome product by Sepharose 4B column. Also, it showed compared to the conventional thin film hydration method, liposomes prepared by reverse phase evaporation would confer more nano-PT encapsulation. (**B**) Nano-Pt-loaded liposomes (Nano-Pt/VP@MLipo), but not free nano-Pt, could be collected through centrifugation (12,000 rpm, 10 min), conferring a simple purification method.

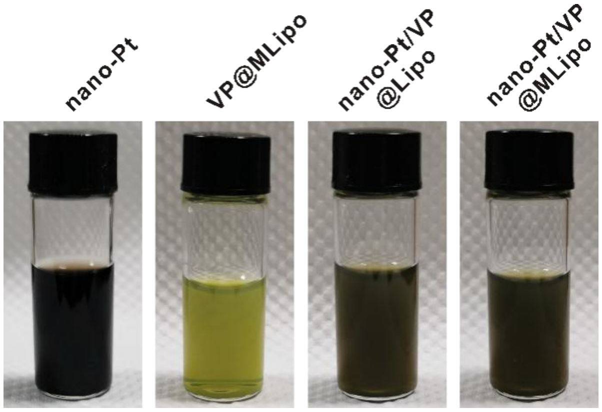

Figure S2. Photographs of free nano-Pt (dark brown), VP@MLipo (yellow green), nano-Pt/VP@Lipo, and nano-Pt/VP@MLipo.

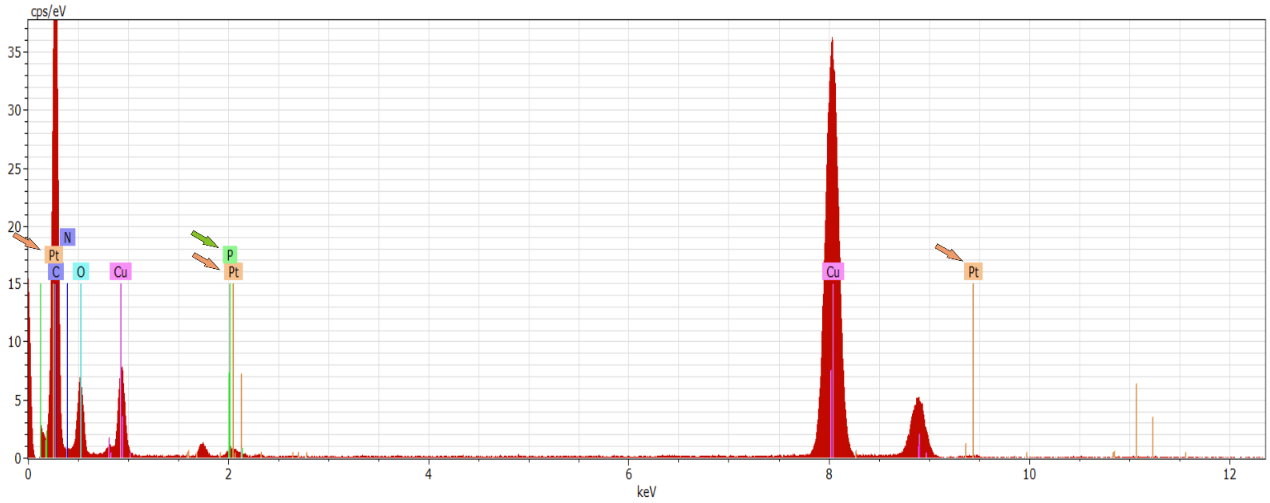

Figure S3. The Energy-Dispersive X-Ray Spectroscopy (EDS) spectrum of nano-Pt/VP@MLipo. The identified Pt (orange arrows) and P (green arrow) signals indicated the specific element compositions of the hybrid liposomes encapsulating nano-Pt.

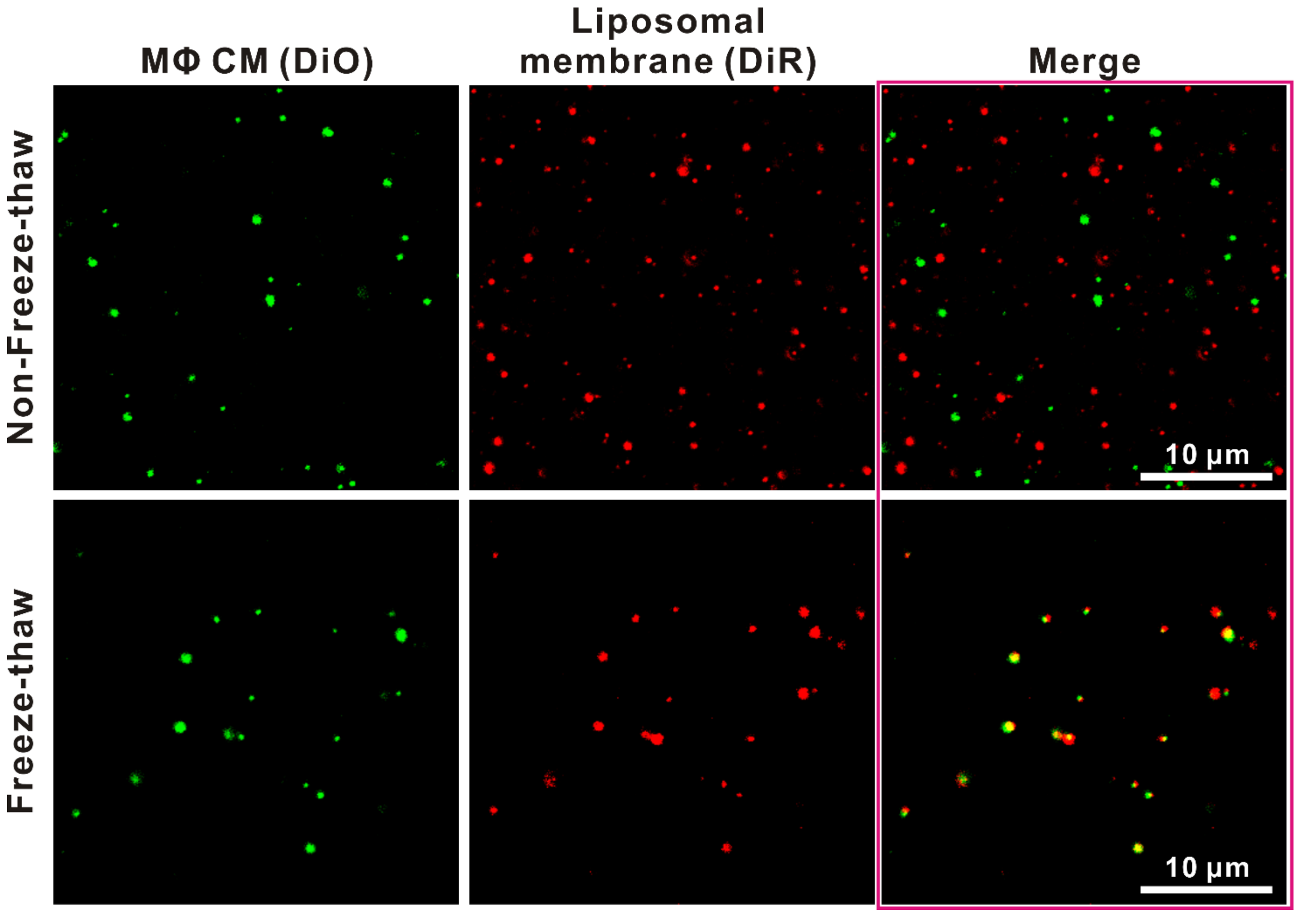

Figure S4. Mφ cell membrane (CM) vesicles and liposomal membrane were labeled with DiO and DiR fluorescent dye respectively. The fluorescence colocalization was obtained only after freeze-thaw cycles. The merge image is also placed in the main text as Fig. 1E.

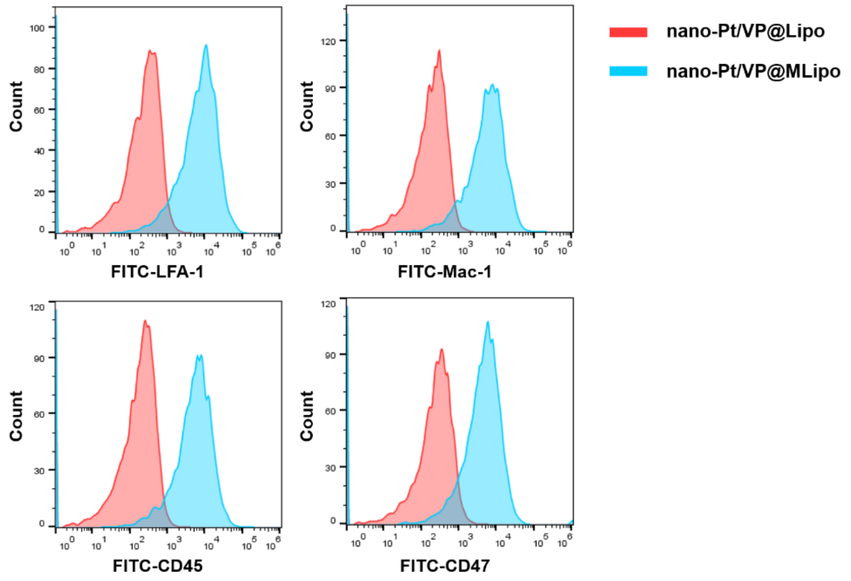

Figure S5. The presence of the LFA-1, Mac-1, CD45 and CD47 and their correct orientation on the nano-Pt/VP@MLipo surface were identified using flow cytometry. Conventional liposomes (nano-Pt/VP@Lipo) were set as control.

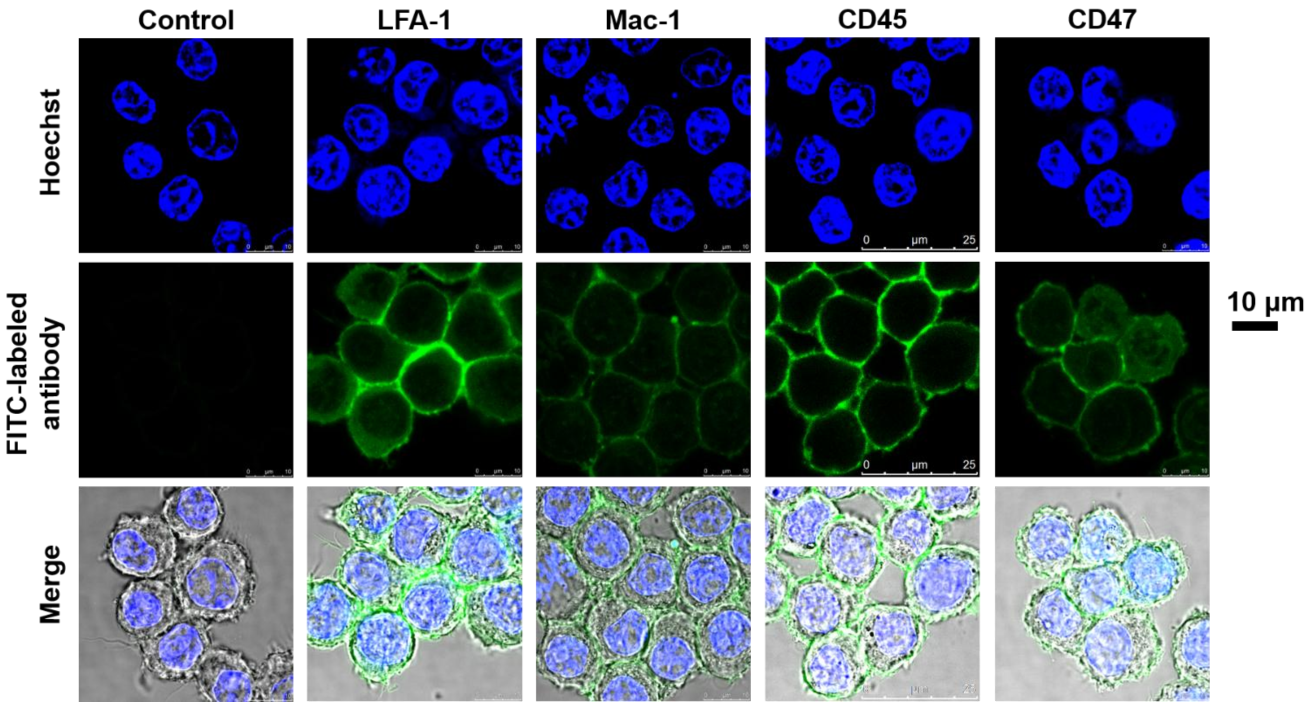

Figure S6. Immunofluorescence analysis of RAW264.7 macrophages stained with FITC-labeled anti-CD45, anti-CD47, anti-LFA-1, or anti-Mac-1, respectively. Cells stained with FTIC-labeled IgG were set as control.

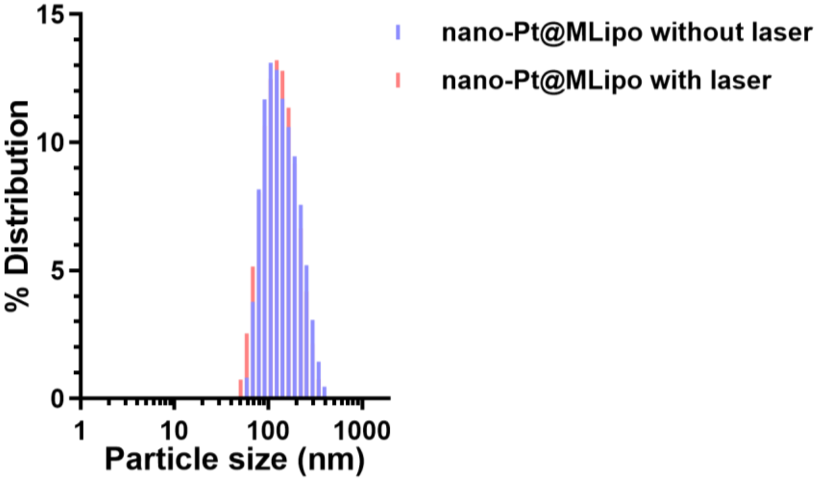

Figure S7. Influence of light irradiation (690 nm, 100 mW/cm^2, 10 min) on the size of nano-Pt/MLipo.

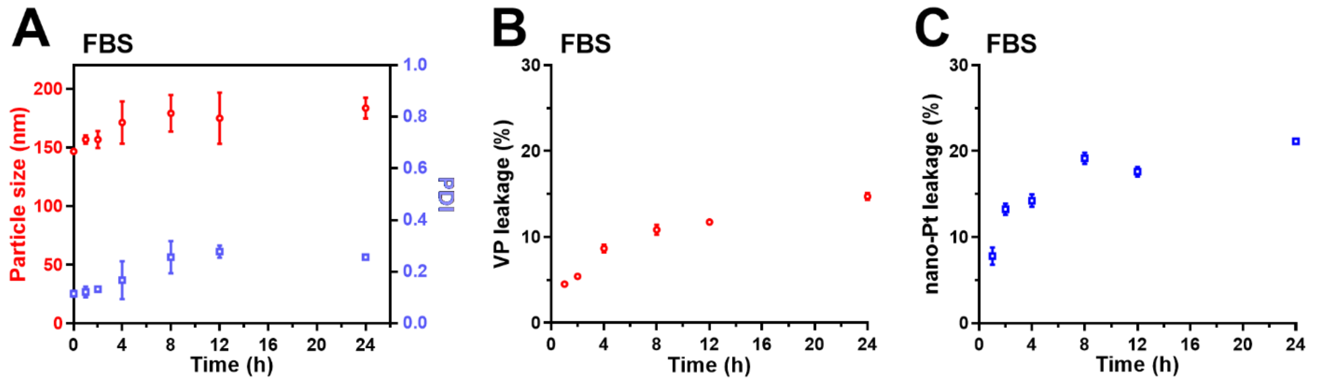

Figure S8. Colloid stability and leakage of nano-Pt/VP@MLipo in FBS at 37 ℃. (**A**) Particle size and PDI. (**B**) VP leakage. (**C**) nano-Pt leakage. Values are presented as mean ± s.d. (n=3). PDI, polydispersity index.

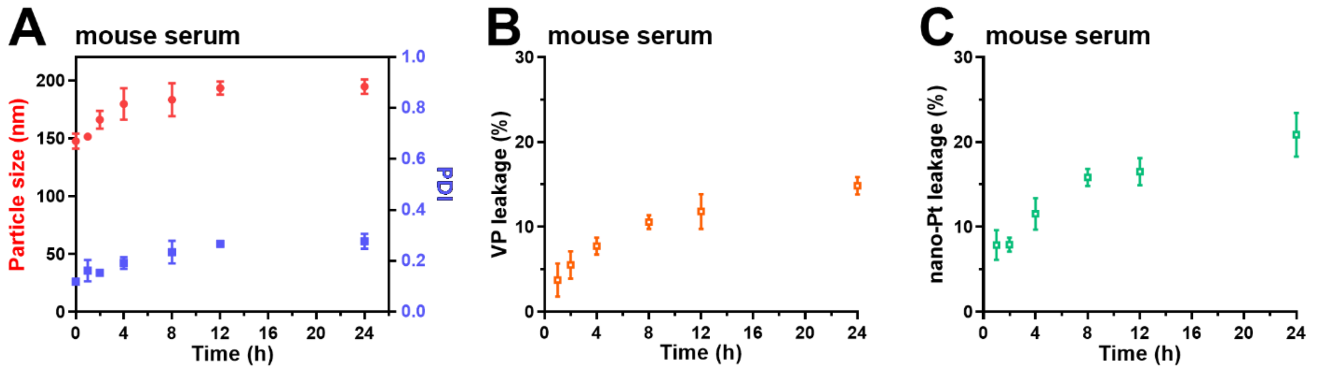

Figure S9. Colloid stability and leakage of nano-Pt/VP@MLipo in mouse serum at 37 ℃. (**A**) Particle size and PDI. (**B**) VP leakage. (**C**) nano-Pt leakage. Values are presented as mean ± s.d. (n=3). PDI, polydispersity index.

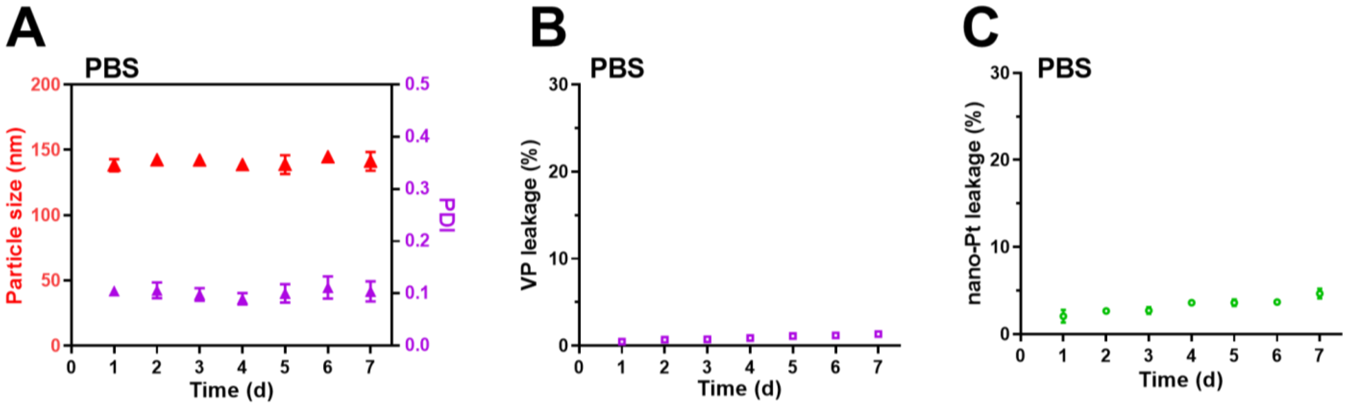

Figure S10. Colloid stability of nano-Pt/VP@MLipo in pH 7.4 PBS at 4 ℃. (**A**) Particle size and PDI. (**B**) VP leakage. (**C**) nano-Pt leakage. Values are presented as mean ± s.d. (n=3). PDI, polydispersity index.

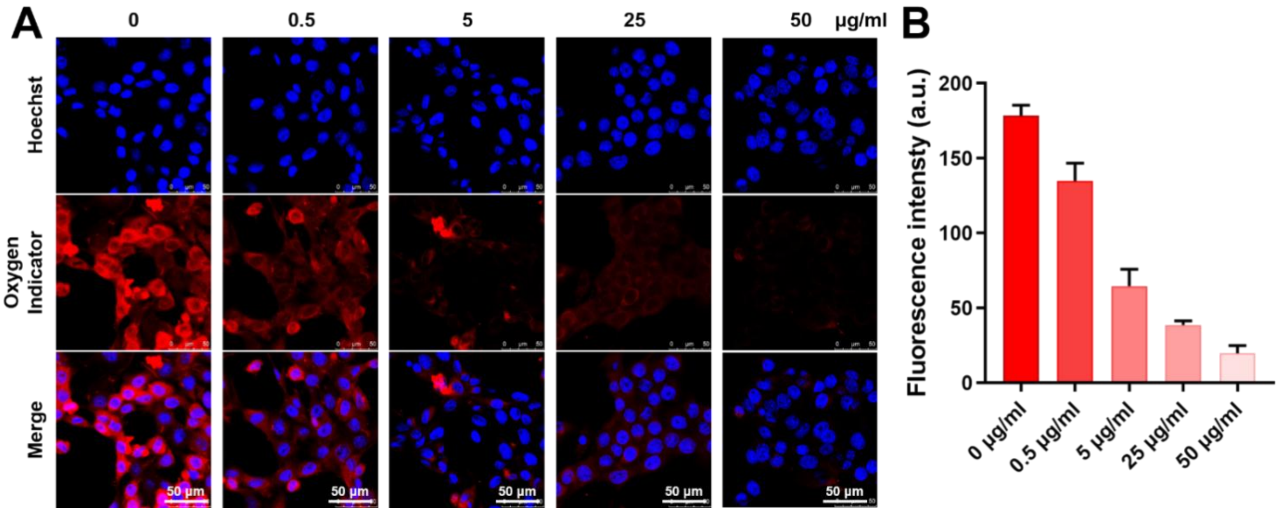

Figure S11. Pt concentration (0~ 50 μg/ml)-dependent oxygen generation in 4T1 cells under hypoxia. (**A**) CLSM images of fluorescent O_2 indicator $(Ru(dpp)_3)Cl_2$. (**B**) Quantified fluorescence intensity. Values are presented as mean ± s.d. (n=3).

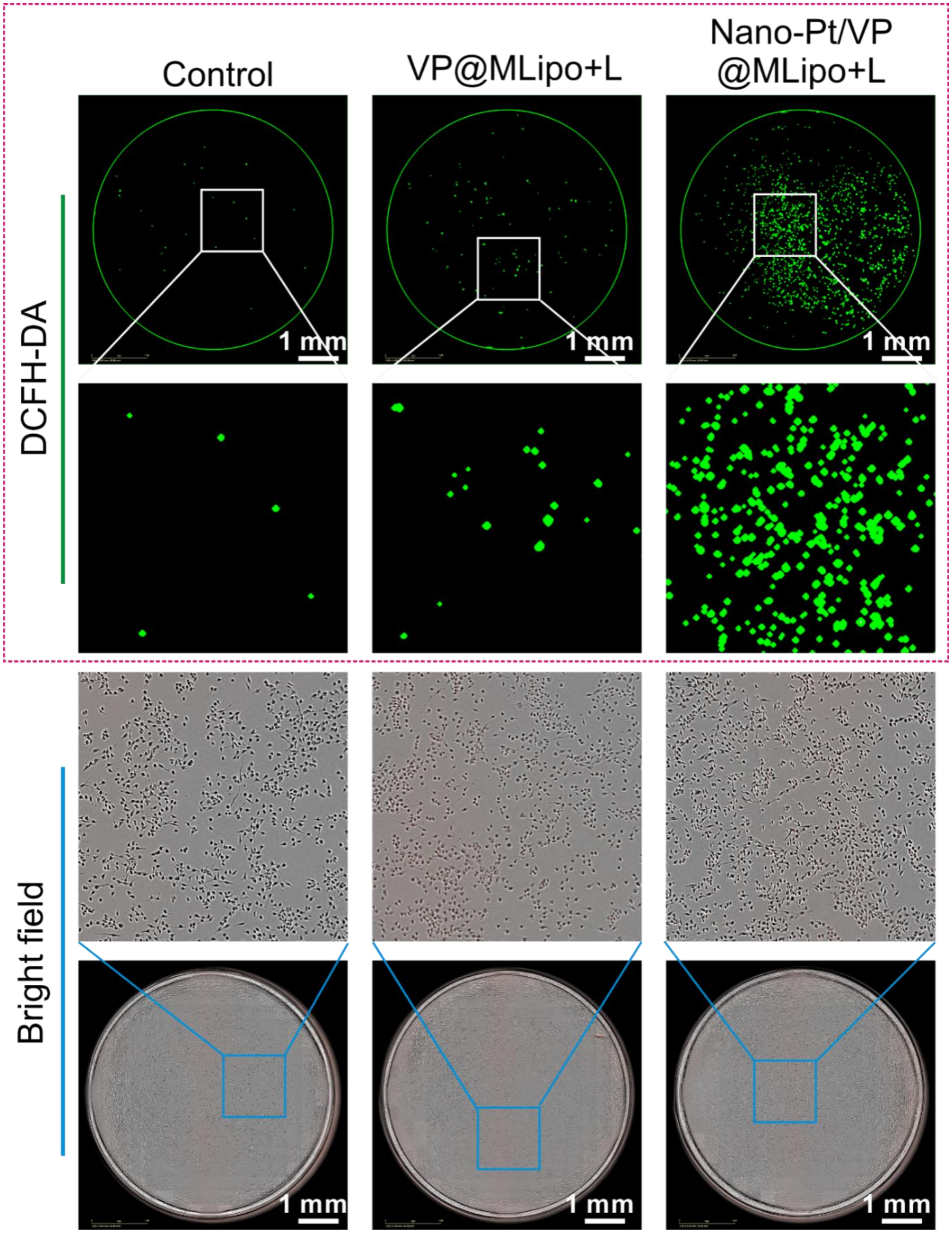

Figure S12. Intracellular ROS production upon light (690 nm) irradiation detected using the probe DCFH-DA. The fluorescent 4T1 cells in the 96-well plate were photographed using IncuCyte live cell analysis system. Both the fluorescent and the corresponding bright field were shown. The part in the dotted box (top two rows) is also placed in the main text as Fig. 2E.

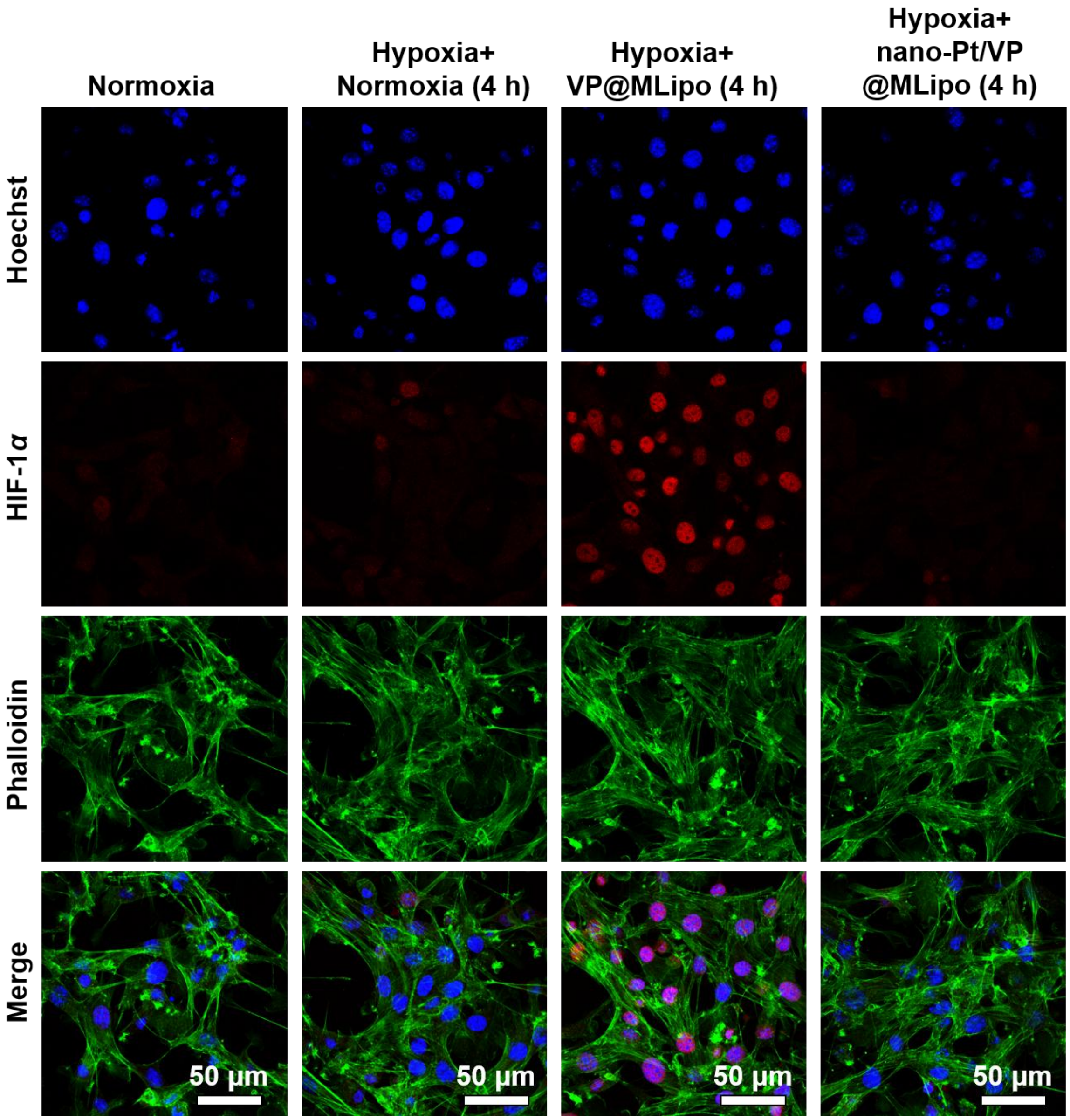

Figure S13. CLSM images of HIF-1*α* expression and its nuclear translocation in 4T1 cells after different treatments.

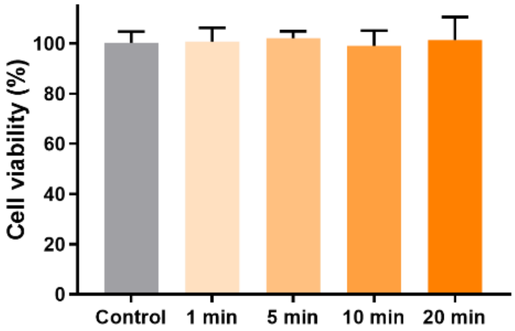

Figure S14. Influence of light irradiation (690 nm, 100 mW/cm^2) alone on cell viability. Different

irradiation durations were tested. Values are presented as mean ± s.d. (n=3).

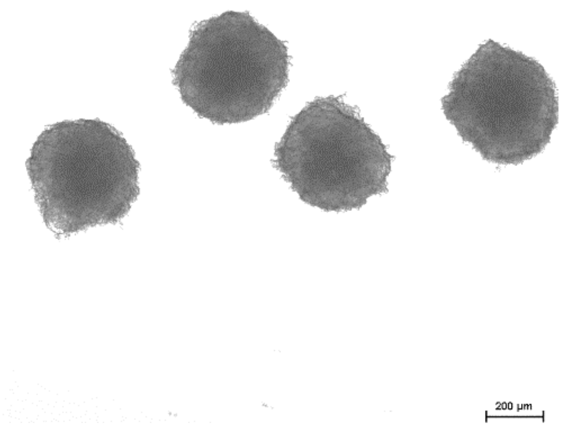

Figure S15. Representative photograph of established 4T1 tumor spheroids.

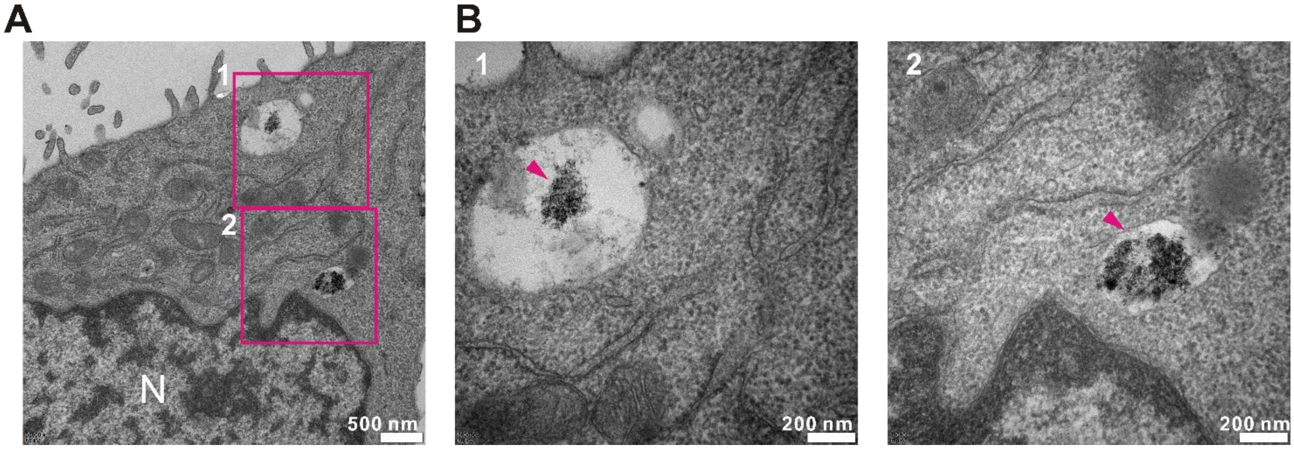

Figure S16. Typical TEM images showed that nano-Pt (indicated with red arrow heads) was internalized in the lysosomes of 4T1 cells at the periphery of the tumor spheroids. The region in the red box (#1 and 2) in **A** are magnified in **B**. N, nucleus.

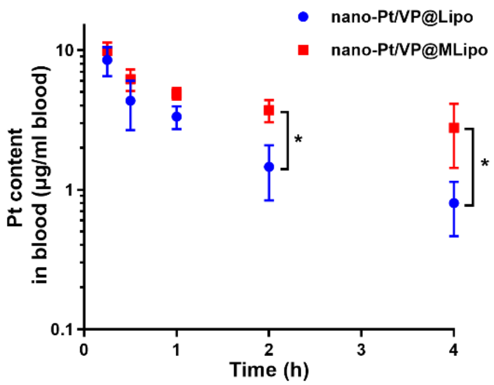

Figure S17. Platinum concentration in blood after i.v. injection of the liposomes. Values are presented as mean ± s.d. (n = 5). *p < 0.05.

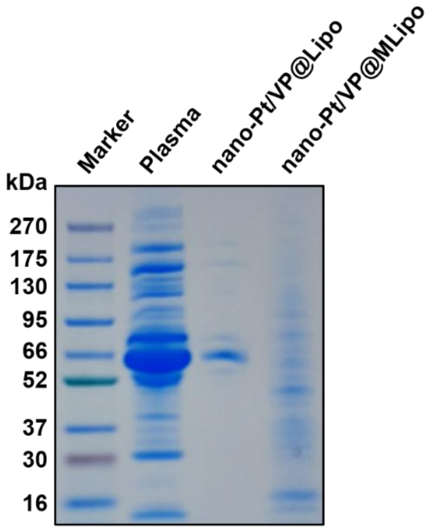

Figure S18. Protein corona assay of the liposomes after incubation in mouse plasma for 4 h at 37 ℃. Noted that as for nano-Pt/VP@MLipo, the detected protein bands may, in a considerable portion, come from Mφ membrane protein due to the Mφ CM-hybridized membrane structure.

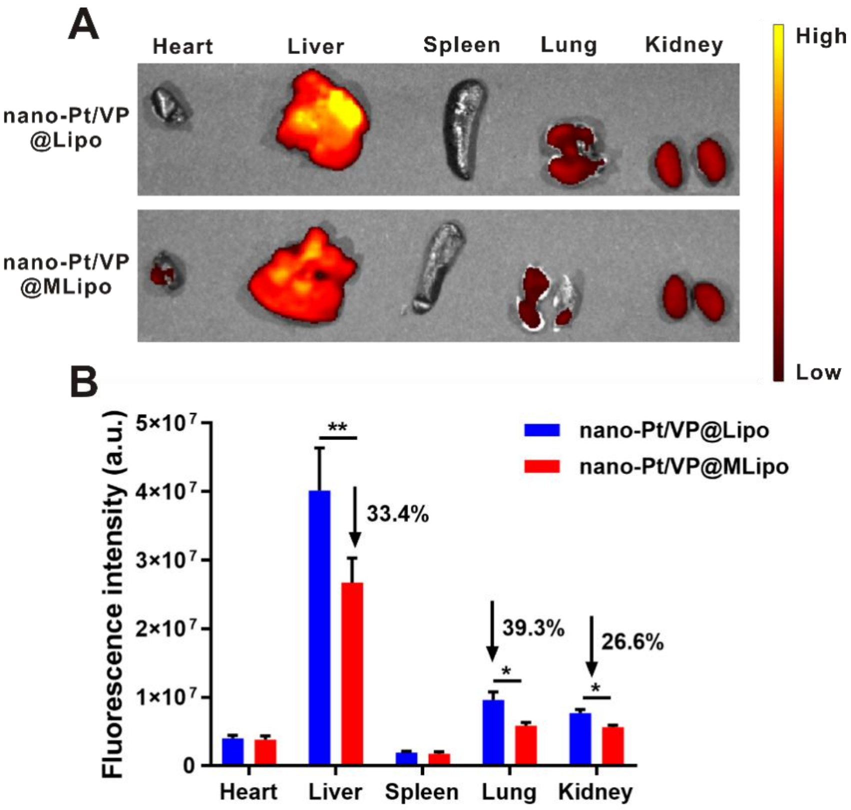

Figure S19. Biodistribution of the liposomes in major organs 4 h after i.v. injection. (**A**) Photos of the organs under IVIS Spectrum CT imaging system. (**B**) Quantified VP fluorescence. Values are presented as mean ± s.d. (n=4). *p < 0.05, **p < 0.01.

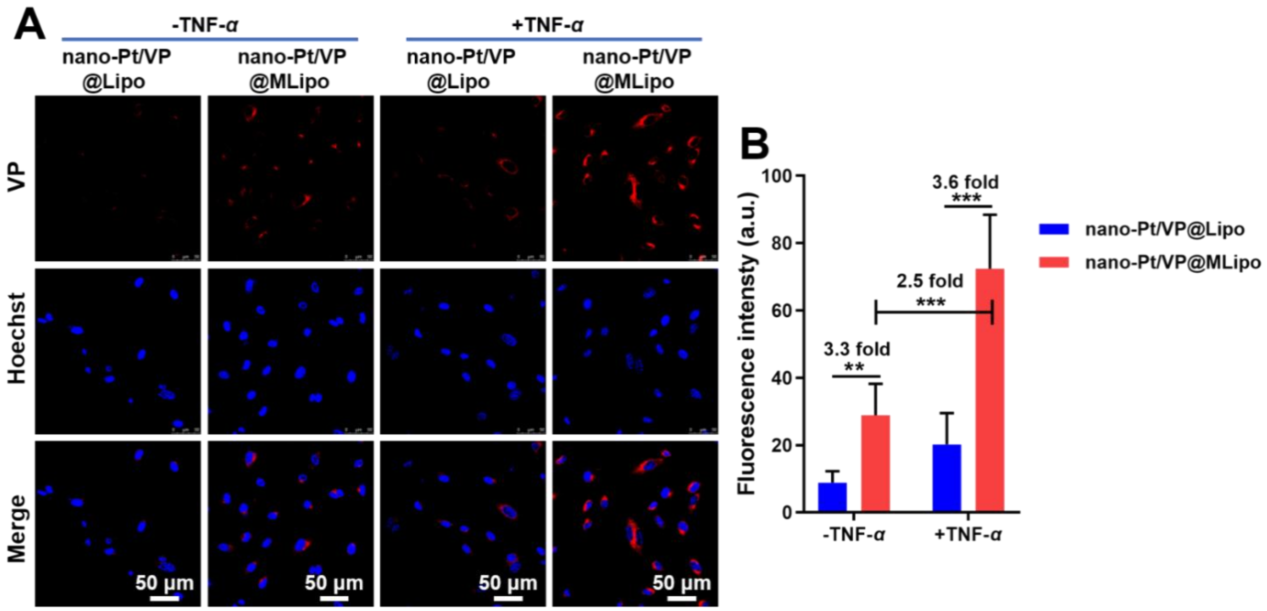

Figure S20. Targeting of liposomes in flow condition to HUVEC cells. The cells were previously treated with TNF-*α* to mimic the inflamed condition as that in solid tumors. (**A**)

Representative CLSM images. (**B**) Quantified VP fluorescence in HUVEC cell. Values are presented as mean ± s.d. (n=3) **p<0.01, ***p< 0.001.

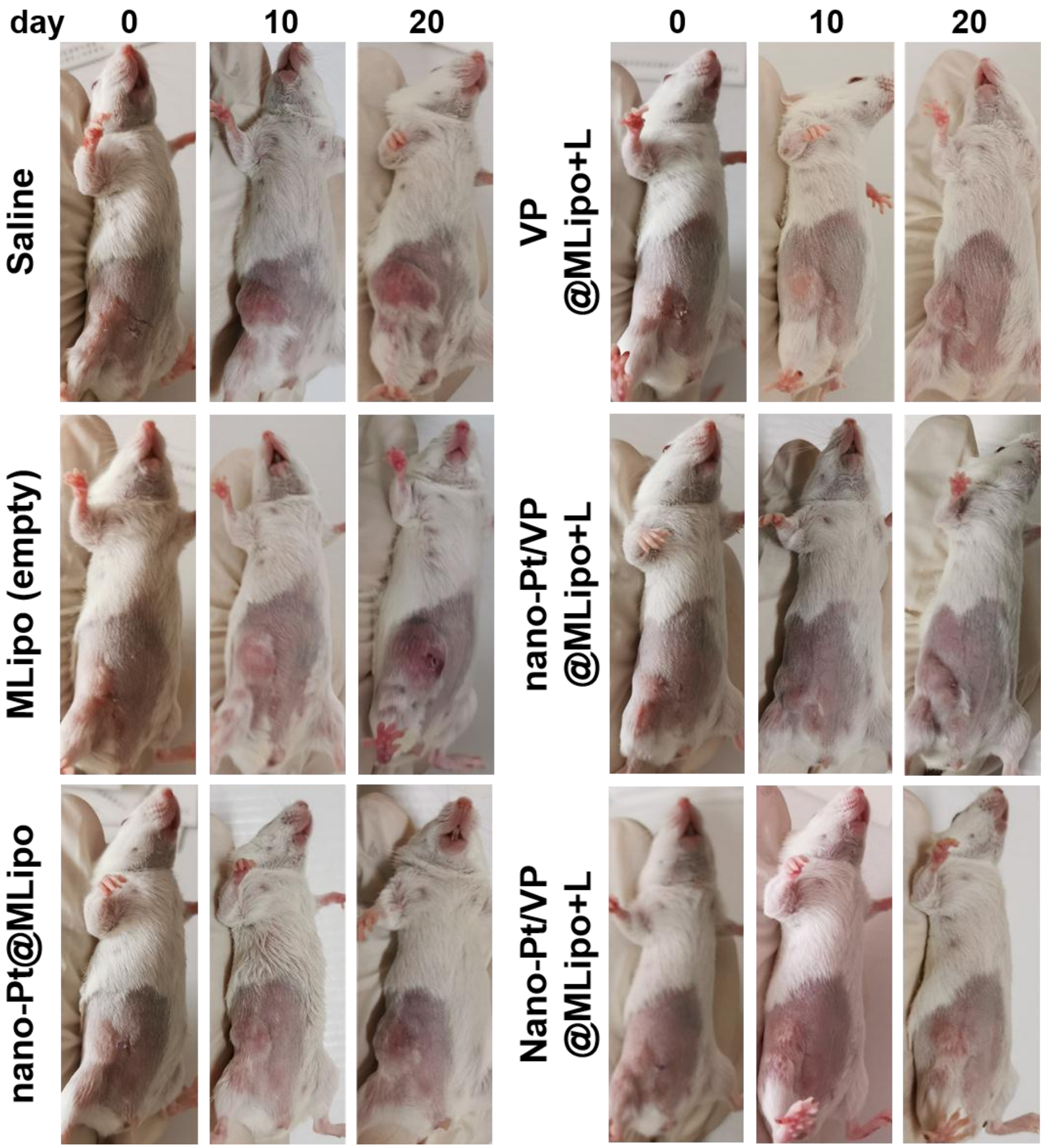

Figure S21. Photos of the 4T1 tumor-bearing mice during the in vivo therapy. One representative mouse from each group on day 0, 10, and 20 is shown.

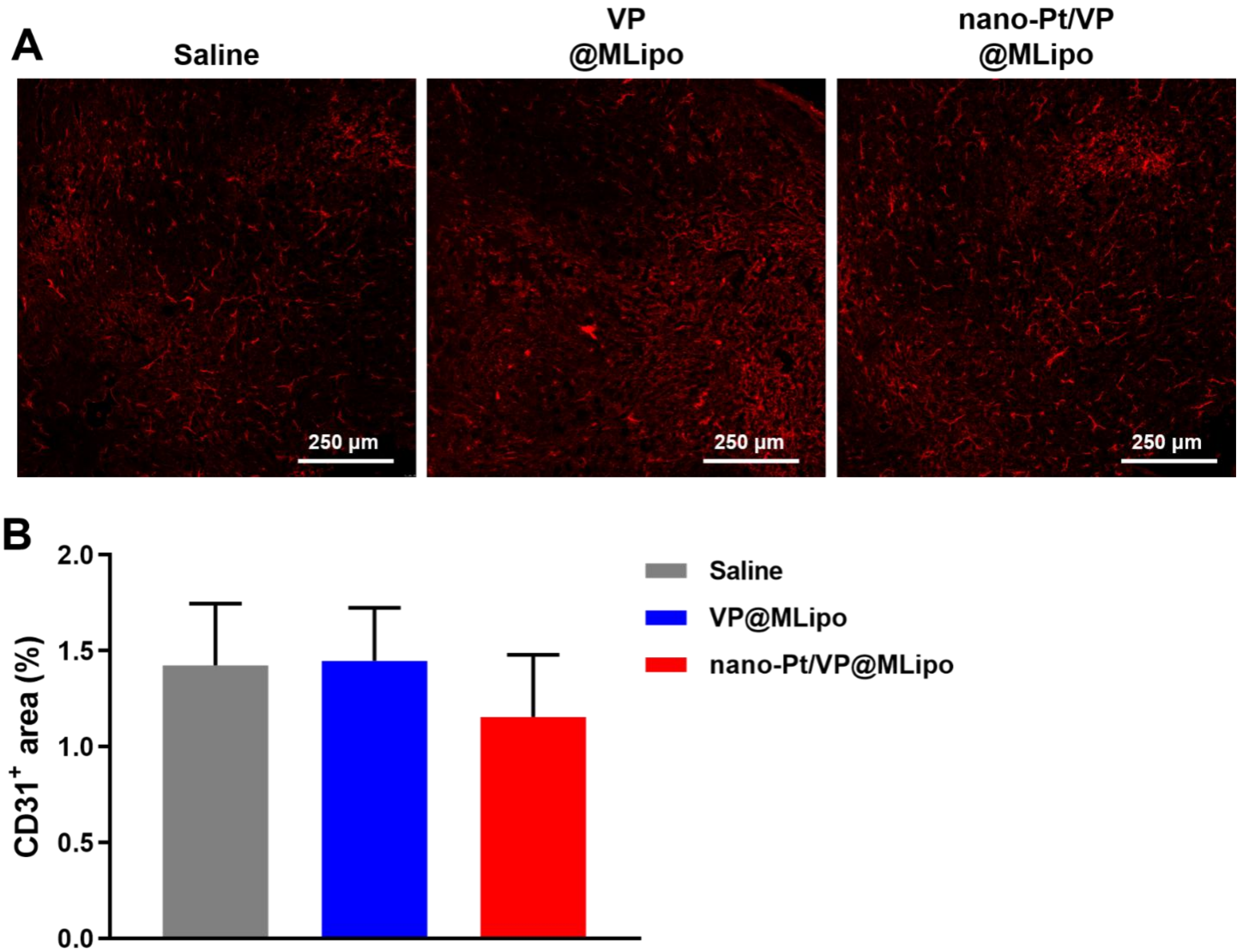

Figure S22. Tumor vessel examination. (**A**) CLSM images of CD31-stained 4T1 tumor vessels of the same field of vision in Figure 6H. (**B**) Quantified vessel area in tumors. Values are presented as mean ± s.d. (n=3)

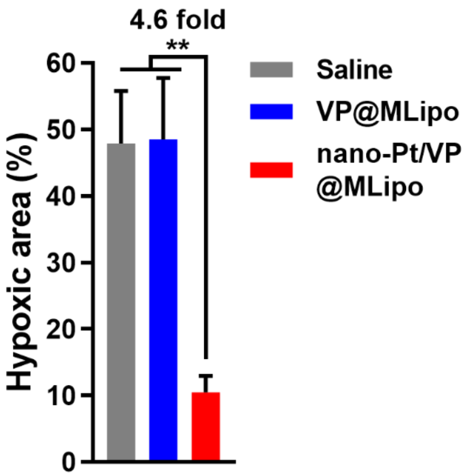

Figure S23. Quantified hypoxic area. Values are presented as mean ± s.d.(n=3). **$p < 0.01$.

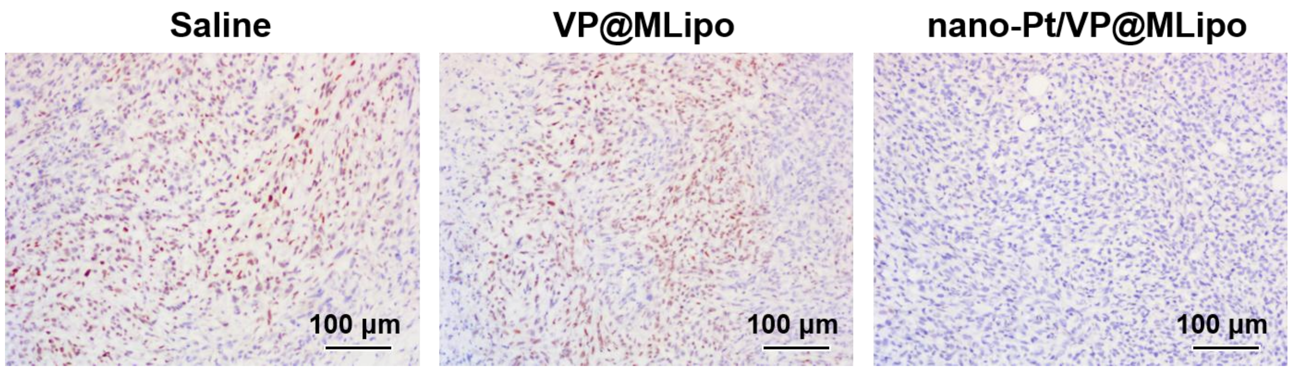

Figure S24. HIF-1α expression (brown) identified through immunohistochemical staining. 4T1 tumors were excised for this assay 4 h after liposome i.v. injection.

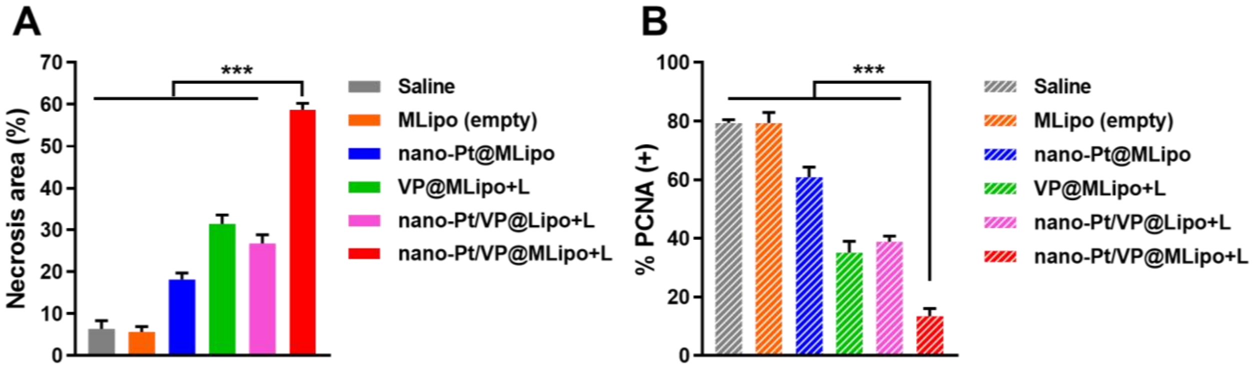

Figure S25. Statistical assay of the necrosis area (**A**) and PCNA positive tumor cells (**B**) in 4T1 tumors after therapy. Values are presented as mean ± s.d. (n = 3). *** $p< 0.001$.

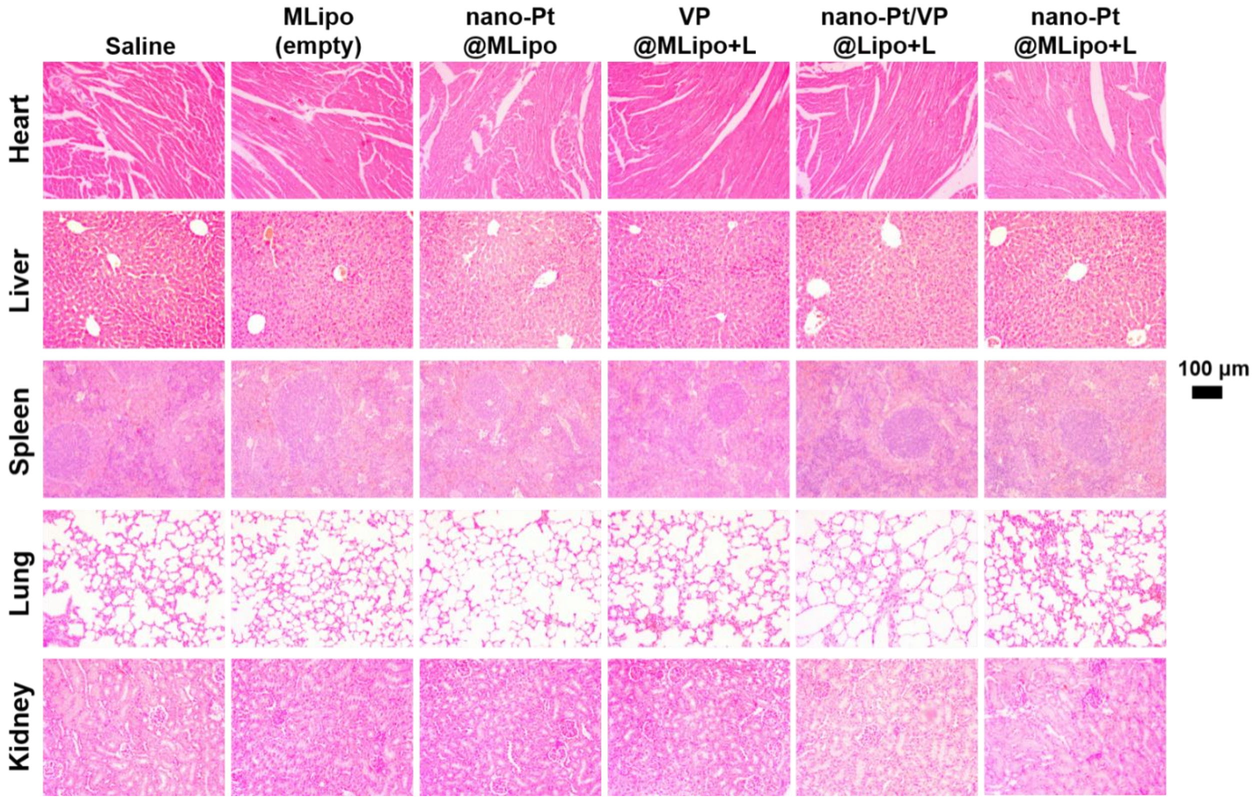

Figure S26. On day 1 (24 h after liposome injection), 3 mice from each group were sacrificed, and the major organs were processed for paraffin sections and histopathological examination (H&E staining).

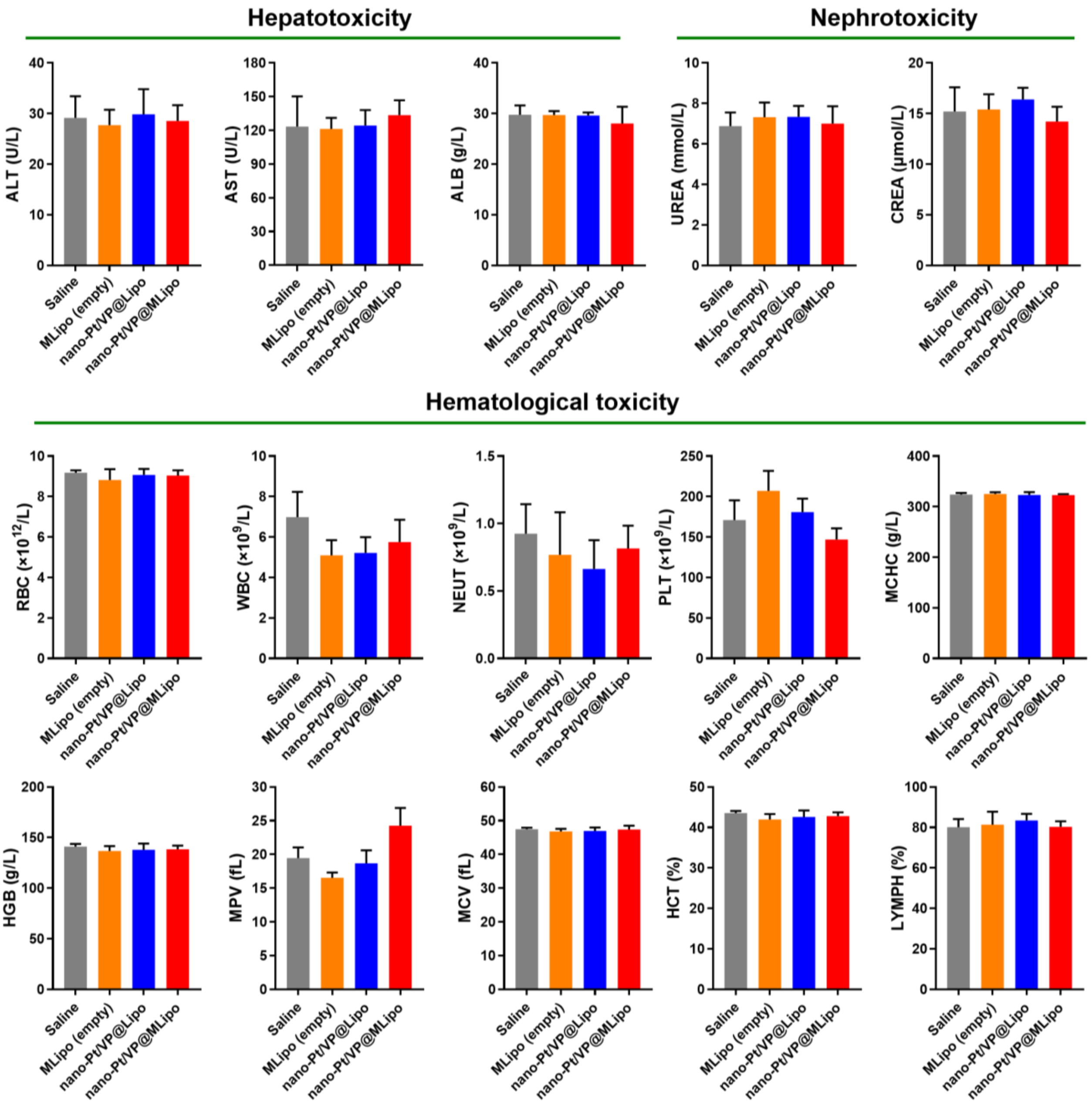

Figure S27. Toxicity profile of nano-Pt/VP@MLipo in healthy female Balb/c mice. The liposomes were i.v. injected to the mice. After 24 h, the blood was collected from the mouse orbital for serum biochemistry and complete blood panel analysis. Four mice were used in each group for this experiment. ALT, alanine aminotransferase. AST, aspartate aminotransferase. ALB, albumin. CREA, creatinine. RBC, red blood cells. WBC, white blood cells. NEUT, neutrophils. PLT, platelets. MCHC, mean corpuscular hemoglobin concentration. HGB, hemoglobin. MPV, mean platelet volume. MCV, mean corpuscular volume. HCT, hematocrit. LYMPH, lymphocytes. Values are presented as mean ± s.d.(n=3).

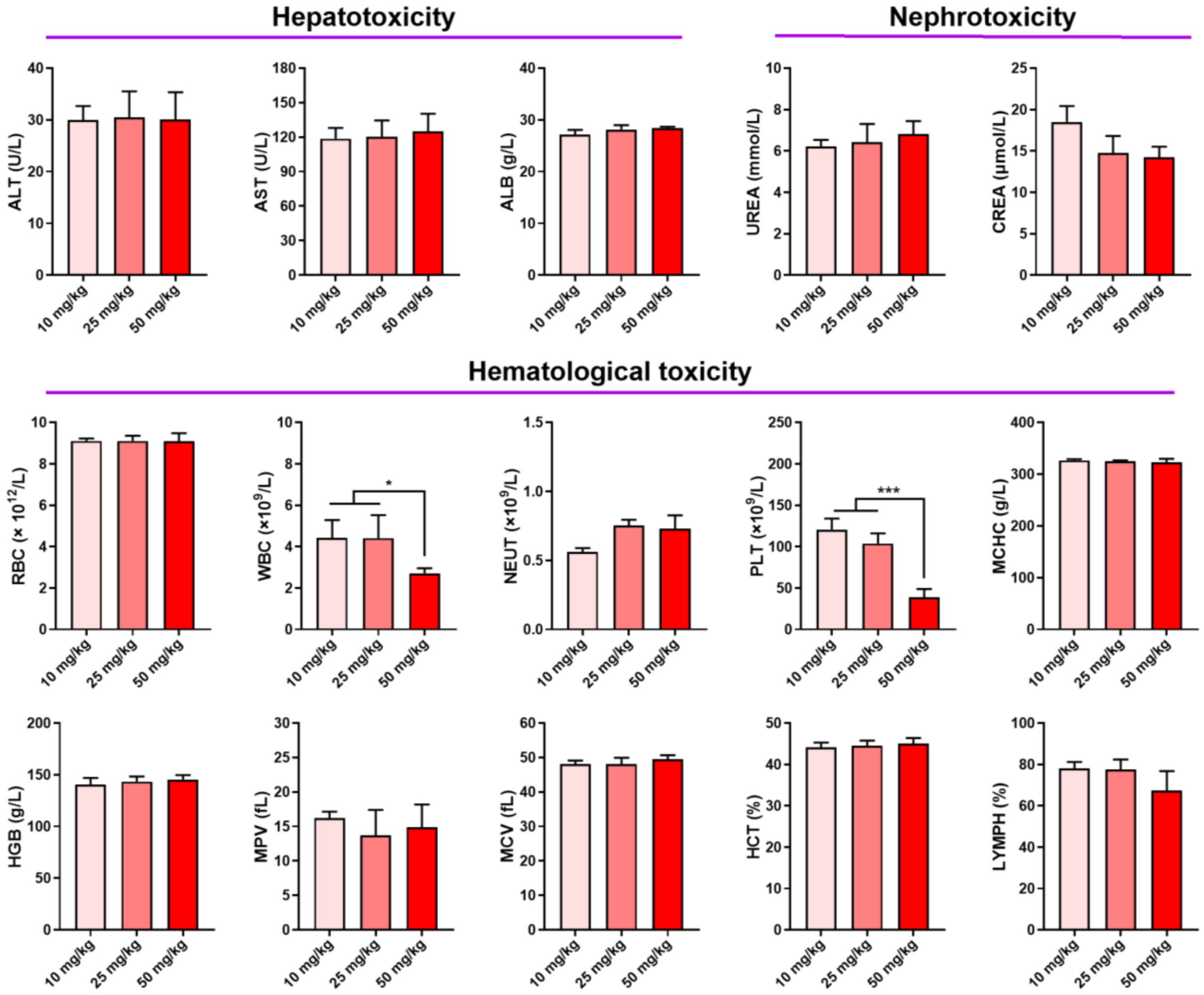

Figure S28. Serum biochemistry and complete blood panel analysis of the mice in the dose escalation test. The dose of nano-Pt (10, 25, 50 mg/kg) of nano-Pt/VP@MLipo was used as the sub-group label as the VP without light was considered to be safe. Values are presented as mean ± s.d.(n=3), *p < 0.05, ***p < 0.001.

Table S2 Noncompartmental pharmacokinetic parameters of VP in nano-Pt/VP@Lipo, VP@MLipo, and nano-Pt/VP@MLipo (n=5).

	$T_{1/2}$ (h)[a]	C_{max} (ng/ml)[b]	AUC_{0-24} (h*ng/ml)[c]	MRT_{0-24} (h)[d]	Cl (ml/h)[e]	V_z (ml/g)[f]
nano-Pt/VP@Lipo	7.8	2358.8	12543.67	10.34	0.41	0.34
VP@MLipo	12.6	2678.9	16690.12	14.25	0.30	0.19
nano-Pt/VP@MLipo	12.3	2612.2	17600.86	13.96	0.29	0.22

[a] $T_{1/2}$, half-life in plasma. [b] C_{max}, the maximum plasma concentration. [c] AUC, area under the curve. [d] MRT, Mean residence time. [e] Cl, clearance. [f] V_z, volume of distribution.

Table S3. Survival analysis of the mice with various treatments.

	Median survival (day)	*ILS* (day) [a]	% *ILS* [b]
Saline	25	-	-
MLipo (empty)	26	1	4
nano-Pt@MLipo	30	5	20
VP@MLipo+L	32	7	28
nano-Pt/VP@Lipo+L	34	9	36
nano-Pt/VP@MLipo+L	43	18	72

[a] *ILS*: Increase in Life Span (T - C), where T and C are the mean survival time of treated mice and control mice from the saline group.

[b] $\%ILS = (T/C-1)\times 100\%$
